# Research Advances in CO_x_ Hydrogenation to Valuable Hydrocarbons over Carbon-Supported Fe-Based Catalysts

**DOI:** 10.3390/molecules30112268

**Published:** 2025-05-22

**Authors:** Shuai Peng, Chao Deng, Lujing Xu, Junli Li, Ruxing Gao

**Affiliations:** 1 School of Energy Science and Engineering, Nanjing Tech University, Nanjing 211816, China; ps040105@163.com; 2 State Key Laboratory of Materials-Oriented Chemical Engineering, College of Chemical Engineering, Nanjing Tech University, Nanjing 211816, China; dengc@njtech.edu.cn (C.D.); lujing@njtech.edu.cn (L.X.); l1289676608@njtech.edu.cn (J.L.)

**Keywords:** CO hydrogenation, CO_2_ hydrogenation, carbon support, Fe-based catalyst

## Abstract

The overconsumption of fossil energy sources has resulted in serious environmental impacts and an ensuing energy crisis. Therefore, the search for a new alternative energy technology has become a focus of attention. The long-established Fischer–Tropsch synthesis technology and the recent CO_2_ hydrogenation technology with unlimited potential seem to be among the ways to solve the above problems. Among them, the development of efficient Fe-based catalysts has become a key issue. Weaker interactions on carbon supports are more favourable for the formation of active phases in Fe-based catalysts than stronger metal–support interactions on conventional oxide supports. In this work, we systematically summarise the application of various types of carbon materials (carbon nanotubes, mesoporous carbon, graphene, activated carbon, etc.) in CO_x_ hydrogenation reactions. The effects of different structural types of carriers on the dispersion of active sites are discussed. At the same time, the effects of different carrier preparation methods on catalytic performance are compared. In addition, the role of surface modifications to carbon materials in the promotion of active sites is discussed. Finally, we propose possible research directions based on the current problems in these catalytic systems. The aim is to provide a reference for the development of new carbon materials and their application in CO_x_ hydrogenation.

## 1. Introduction

With the progress of science and technology, human society is developing at a high speed. In recent years, mankind’s dependence on fossil energy has become more and more obvious, and the annual global consumption of crude oil is increasing. This means that human society will eventually face the depletion of fossil energy, and the search for alternative energy has become the key to alleviating this energy crisis. Therefore, the rationalisation of the use of gases such as carbon dioxide, carbon monoxide, and methane has recently become a hot topic. On the one hand, the oxidation of CO can be used for both electrical/thermochemical energy storage and the generation of CO_2_ for easy capture, contributing to carbon emission reduction targets [1,2]. On the other hand, obtaining high-value-added hydrocarbon products through hydrogenation is also an effective measure to save energy and reduce emissions.

The Fischer–Tropsch synthesis (FTS) technique, developed by German chemists in 1925, successfully converts syngas (CO + H_2_) into hydrocarbons, and this technology has been successfully industrialised for nearly a century [3]. Technology can use syngas to produce a wide range of chemicals such as low-carbon olefins, gasoline, diesel, paraffin, etc., while the products can also be used as chemical feedstocks to produce fibers, rubber, and surfactants, among others. The industrialisation of this technology has contributed greatly to alleviating the consumption of fossil energy. Not only that, but the massive use of fossil energy has caused the concentration of CO_2_ in the air to rise year after year. The large amount of CO_2_ emissions has caused a series of environmental problems, such as the greenhouse effect. Therefore, reducing CO_2_ emissions has become another issue in the process of fossil energy use. Currently, the means of CO_2_ emission reduction include the following two directions: capture and storage and utilisation, and the use of CO_2_ as a carbon source and its conversion into valuable compounds have attracted widespread attention [4]. It has been found that CO_2_ can be converted to CO via the reverse water–gas shift reaction (RWGS), and then to hydrocarbons via the FTS reaction [5]. Therefore, the CO_2_ hydrogenation reaction is also known as an improved FTS reaction. This process is not only an effective way to cope with the energy crisis, but also reduces CO_2_ emissions [6]. The development of efficient and stable catalysts has become the key to this technology.

Both CO and CO_2_ hydrogenation are typically thermocatalytic conversion processes, and Fe and Co are widely used as catalysts in laboratories and factories due to their cheap and easily available properties [7]. However, Fe or Co alone as catalysts do not exhibit significant activity during the reaction due to their lower dispersion. Because the FTS process is a structure-sensitive reaction, the size distribution of metal nanoparticles is closely related to catalytic performance [8,9,10,11]. In response, researchers loaded active components on the surfaces of different types of supports and used the large specific surface areas of the supports to distribute the metals [12]. The particle size of the active sites was modulated to expose more available sites. The results were as expected, showing that the pores of the supports could well inhibit the agglomeration of metal particles and the sintering phenomenon during the reaction, significantly enhancing the catalytic activity. Typical supports include SiO_2_, Al_2_O_3_, TiO_2_, and MgO, which are thermally stable during the reaction [13,14,15]. However, strong interactions between metals and supports may form non-reducible mixed oxides, such as CoSiO_2_, Co_2_AlO_4_, and CoTiO_4_, which prevent the metals from being converted into active sites during the reaction, limiting the catalyst performance to a large extent [6,16].

In recent years, carbon materials have been widely used as various catalyst supports due to their rich pore structure and excellent thermal stability. At the same time, their surface modifiability also plays an important role in regulating their performance in the field of catalysis. The above advantages make them very suitable as supports for CO_x_ hydrogenation catalysts, and they are widely used in CO_x_ hydrogenation reactions [17,18]. Compared with traditional oxide supports, the interaction between metal sites and carbon materials is weaker, which can well promote the reduction and carbonisation process of active components [19,20]. Cheng et al. investigated the effect of silicon and carbon carriers on the structure and performance of catalysts for the synthesis of Fe. The higher degree of carbonisation on the carbon carriers also clearly indicated that weaker metal–carrier interactions favoured the reduction of metal particles [21]. Moreover, as the surface of the carbon-based support can be easily modified, the introduction of heteroatoms or functional groups can reasonably regulate the intensity of the MSI effect and achieve the modulation of the catalyst performance [22,23]. Non-negligibly, introduced heteroatoms and reactive groups can provide dispersive anchor localisation sites and electron donors for active components, further optimising the catalytic performance [24,25,26]. Nitrogen exists in carbon materials mainly in the form of pyridine nitrogen, pyrrole nitrogen, graphitic nitrogen, and pyridine nitrogen oxides, and can be produced by post-doping or in situ doping. Simple preparation methods and excellent catalytic properties make nitrogen atoms stand out among many heteroatoms [27]. Common carbon supports include activated carbon (AC), carbon nanotubes (CNTs), mesoporous carbon, and graphene. In addition, carbon supports derived from MOFs and biomass as precursors have also attracted much attention, and these supports are particularly suitable for use as catalytic supports due to their high specific surface area.

In the CO_x_ hydrogenation reaction, different metal catalysts appear, including Cu, Co, Zn, Fe, etc., among which Fe is widely used for its lower price and wider product distribution [4,28]. In fact, only Co and Fe catalysts are currently used commercially. Fe is widely used in CO_2_ hydrogenation because of its strong olefin selectivity and water–gas shift activity compared to the strong methanation reaction of Co [29]. For FTS and CO_2_ hydrogenation reactions, researchers have carried out extensive studies on composite metal oxide catalysts, but relatively few studies have been carried out on catalysts loaded with carbon-based materials. However, existing studies show that carbon-based materials are well suited for FTS and CO_2_ hydrogenation reactions, and weaker metal–support interactions seem to be more favourable for reduction, producing a carbonated Fe phase, which is considered to be the active phase for the FTS reaction. Thus, carbon-based materials have good potential [30,31]. Previously, the application of carbon materials in CO_x_ hydrogenation reactions has been summarised by some researchers, but the focus of attention in the published literature is on the comparison between carbon supports and conventional oxide supports, highlighting the advantages of carbon materials as catalyst supports. There is no mention of the specific effects of the carbon support type, material preparation method, and surface modification on the reaction. Therefore, this review begins with a review of different types of carbon materials (carbon nanotubes, mesoporous carbon, graphene, activated carbon, carbon spheres, and biomass- and MOF-derived carbon materials) in CO hydrogenation, and CO_2_ hydrogenation reactions are categorised and summarised to analyse the effects of different structures on these reactions; secondly, the review focuses on changes in the physical and chemical properties of the supports due to the methods of material synthesis and preparation, as well as the effects on catalytic performance; finally, methods and approaches to the surface modification (functional groups and heteroatom doping) of carbon materials are sorted to analyse the effects on the reaction in the microenvironment around the reactive site. This study aims to provide a reference for the development of new catalysts with a high efficiency, stability, and greenness.

## 2. CO Hydrogenation

**Figure 1:**
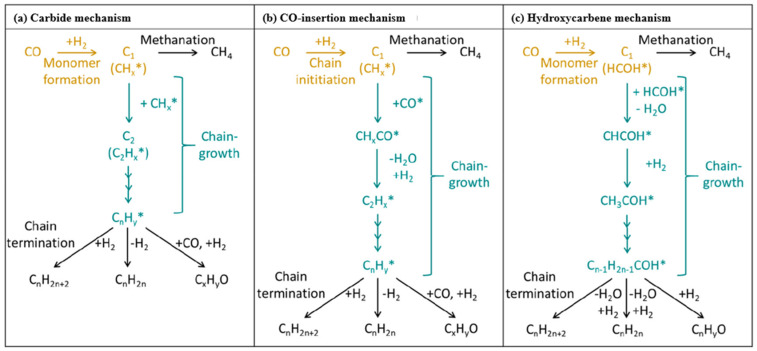
The three most common chain growth mechanisms in FTS are: (**a**) carbide mechanism, (**b**) CO-insertion mechanism, and (**c**) hydroxycarbene mechanism [3]. Copyright (2023) American Chemical Society.

The rapid development of society has been accompanied by an increase in the demand for chemicals, and the massive consumption of fossil energy has triggered human thinking about the development of alternative energy technologies. Among them, Fischer–Tropsch synthesis (FTS) is an important process for converting synthesis gas (CO/H_2_) into high-value-added hydrocarbons. It follows a surface polymerisation mechanism, leading to a hydrocarbon distribution that follows the Anderson–Schulz–Flory distribution, which has led to the proposal of various chain growth pathways, the most prominent of which are the carbocation mechanism, the CO insertion mechanism, and the hydroxycarbene mechanism (as shown in Figure 1) [3].

Iron-based catalysts show unique advantages in FTS, with abundant reserves and low prices, and the product distribution can be adjusted by flexibly adjusting the reaction conditions. They are mainly used for the production of gasoline and linear low-molecular-weight olefins in HT-FTS and long-chain paraffins in LT-FTS [32,33]. In addition, iron-based catalysts can be modified to obtain liquid fuels such as high-octane gasoline and diesel with a high selectivity [34]. It is worth noting that FTS is a typical structure-sensitive reaction, and the performance of iron-based catalysts is largely affected by the type of support used. Different supports and loading modes affect the dispersion of metal nanoparticles and metal–support interactions, which, in turn, affect the FTS activity and product distribution [35,36].

Conventional oxide materials such as Al_2_O_3_, SiO_2_, and TiO_2_ have been widely used as catalyst supports due to their excellent thermal stability and have achieved a good catalytic performance. It is worth noting that during high-temperature roasting, such supports can form some mixed compounds with highly dispersed Fe nanoparticles that are difficult to reduce, resulting in the loss of the active phase. In contrast, the surface inertness and rich pore structure of carbon materials are particularly prominent; additionally, the surfaces of carbon materials have a flexible modifiability, which can be adjusted to adapt to different chemical reaction environments by adjusting their types and quantities, ultimately achieving the role of regulating the MSI effect [35,37]. Most of the current literature revolves around some traditional carbon supports, such as carbon nanotubes, graphene, and mesoporous carbon, including the characterisation of their physical properties (surface area, pore size distribution, etc.), their effect on metal particles in FTS, and comparisons of their activity and stability [35,38,39].

### 2.1. Carbon Nanotubes

After graphite nanofibers were first reported, there was a boom in research on fibrous carbon. The introduction of multi-walled carbon nanotubes (MWNTs) and single-walled carbon nanotubes (SWNTs) has made carbon nanotubes one of the hottest topics in nanoscience [40]. Carbon nanotubes are extremely small in size, with a good electrical conductivity, mechanical strength, and elasticity [19,41]. In catalytic reactions, carbon nanotubes confine the reaction intermediates in the nano-channels, thus prolonging their contact time with the catalyst [42]. Due to these unique and excellent properties of carbon nanotubes, they have been widely used as catalytic supports for CO_x_ hydrogenation in recent decades [43,44,45].

Duan et al. prepared FeK-OX catalysts by the direct redox of K_2_FeO_4_ with CNTs and heat treatment, and the higher chain growth probability, high selectivity of light olefins, and olefin/alkane ratios obtained from their experiments likewise demonstrated the high activity of the catalysts, which, according to TEM, showed more homogeneous nanoparticles with a smaller particle size. Raman spectroscopy also suggested that the sample contained more defects, which favours a high dispersion of Fe particles [46]. Notably, the team also prepared FeK-IM and Fe-IM catalysts by conventional impregnation, and comparative results showed that FeK-OX exhibited a 70.4% hydrocarbon selectivity and better stability, mainly due to the emergence of more defects on the surface of the CNT acting as anchor loci and stabilising the Fe nanoparticles [46].

**Figure 2:**
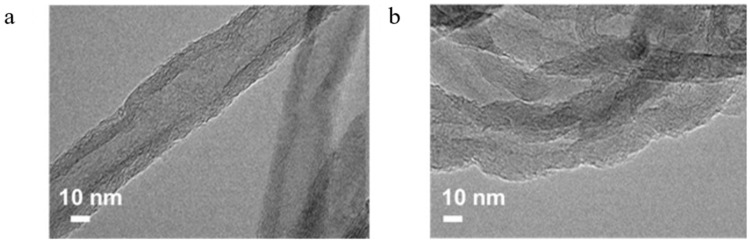
Typical TEM images of CNT (**a**) and CNT-D (**b**) [49]. Copyright (2021) Elsevier.

**Figure 3:**
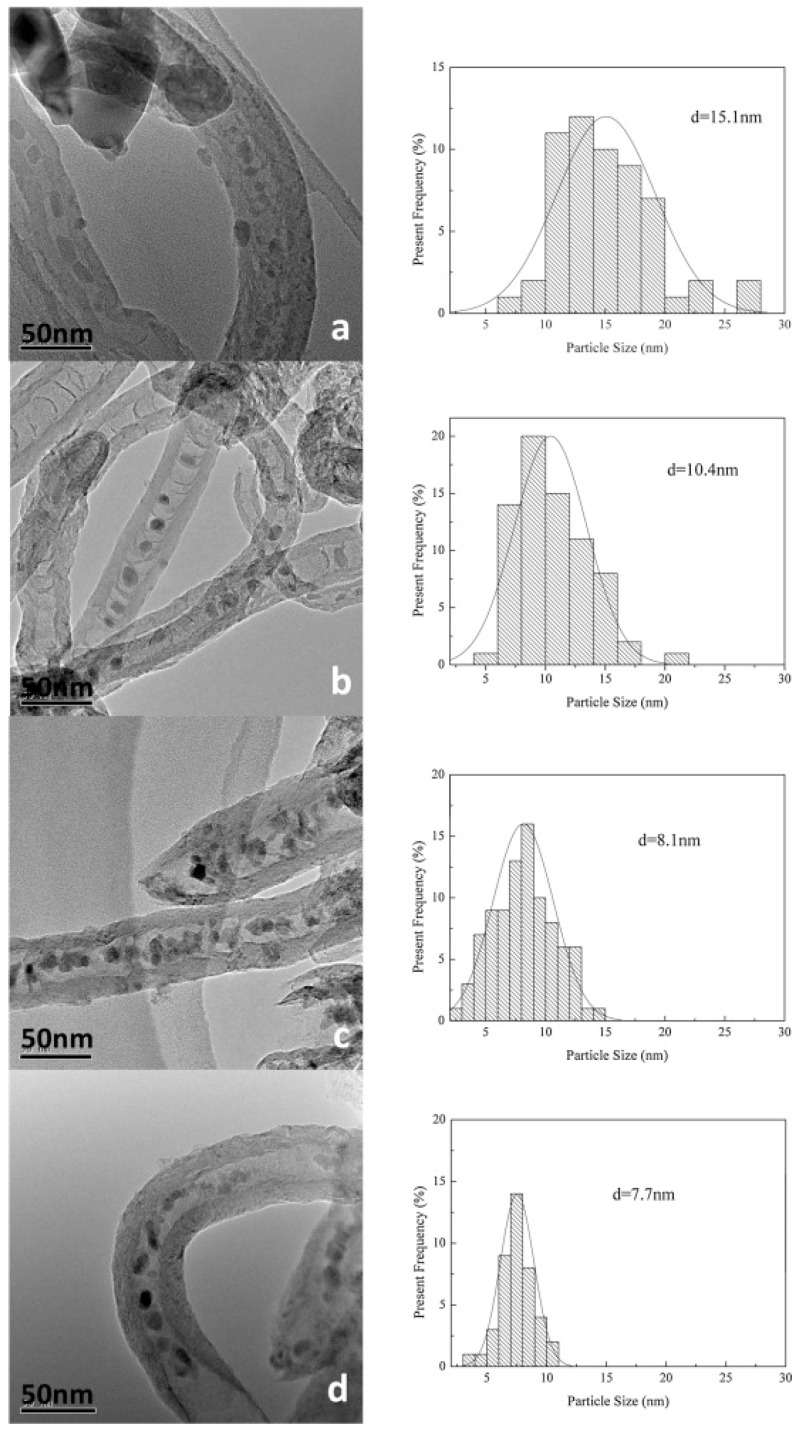
TEM images and histograms of particle size distribution of catalysts: (**a**) Fe/NCNT, (**b**) Fe/NCNT-5, (**c**) Fe/NCNT-10, and (**d**) Fe/NCNT-15 [50]. Copyright (2015) Elsevier.

It should be noted that carbon nanotubes are hydrophobic and chemically inert by nature, which makes it difficult to achieve a high degree of dispersion of metal particles, so it is necessary to carry out surface modification and functionalisation, which can generally be generated through oxidation or nitrogen doping to generate oxygen-containing and nitrogen-containing groups to act as particle anchors [47]. Yahyazadeh et al. compared the catalytic performances of CNT-loaded iron catalysts from different sources. The synthesised CNTs were prepared by chemical vapor deposition (CVD). The experimental results showed that the catalysts prepared by this method had a larger pore volume and pore size than commercial ones, and that the CNTs under this acid pretreatment exhibited more defects and anchor localisation sites, which mitigated the agglomeration of iron oxide particles [48]. Compared with Al_2_O_3_ carriers, CNT carriers exhibited higher CO conversion and light olefin selectivity despite their lower C_5+_ selectivity, and the smaller Fe_2_O_3_ particle size on CNT carriers was more favourable for the contact between active sites and syngas. To improve the surface roughness of the support and shorten the induction period, Fang et al. introduced more defective sites by treating commercial CNTs with a mixture of acids (as shown in Figure 2). The calculated increase in the I_D_/I_G_ values also matched the results of the TEM images, and H_2_-TPR showed that the acid treatment significantly reduced the reduction temperature of the iron species, which resulted in a shorter induction period and improved the initial catalytic activity [49]. Only then did XRD patterns show that the modified CNTs promoted the generation of the active phase χ-Fe_5_C_2_ and increased the FTO activity. Li et al. treated an in situ doped N-CNT with a bamboo-like structure with different concentrations of acid, which led to the ring-breaking of the pristine structure and the generation of a large number of oxygen-containing groups [50]. TEM images showed that the low concentration of acid could not completely destroy the dendritic structure, which also prevented the formation of Fe_2_O_3_ particles in the CNT tubes, and the dispersion of Fe oxides was enhanced with an increase in the acid concentration and the particles gradually shifted to smaller sizes (as shown in Figure 3). According to the XRD analysis, the graphitisation of the acid-treated NCNT was intensified, which facilitated electron transfer and CO activation [50]. The experimental results showed that the acid-treated catalysts exhibited a high C_5+_ selectivity up to 76.5%, which suggested some contributing factors, such as electron transfer, a high dispersion of iron oxide providing more active centres, and iron oxide particles inside the NCNT enhancing CO chemisorption and dissociation [50,51,52].

Xiong et al. prepared N-CNTs at different temperatures by a post-doping method, TEM images showed that above 850 °C, the surfaces of the N-CNTs showed a spherical carbon shape and the internal pore cavities contracted. Raman spectroscopy showed that there was a decreasing tendency of I_D_/I_G_ at 700–850 °C, and that the N doping enhanced the graphitisation of the carbon material, while it increased significantly at 900 °C, indicating that too much N instead broke the ordering of graphene layers [53]. In addition, the team obtained different catalysts by pretreatment with different acid concentrations and temperatures. TEM images showed that FeO_x_ had a large particle size under mild conditions, whereas it was relatively smaller, more narrowly distributed, and functionalised to produce more surface groups under harsh conditions, which explains the higher FTS activity and C_5+_ selectivity exhibited under harsh conditions [53]. Chew et al. prepared catalysts with different surface functional groups using ammonium ferric citrate as the iron source, experimentally demonstrating that functional groups with a high thermal stability could reduce the sintering of iron carbide nanoparticles and improve the stability of the catalysts [54]. Overall, the dispersion of metal particles could be promoted by nitrogen doping or other co-processing methods, and the unique electronic effects and additional surface groups also improved the activity of catalytic reactions and the stability of catalysts to a certain extent. Interestingly, the Fe/NCNT catalyst had a higher CO conversion and chain growth probability in the above experiments, which was likely due to the fact that the nitrogen-containing functional groups were more stable at high temperatures, whereas the decomposition of the oxygen-containing functional groups also resulted in the dislodging of Fe nanoparticles to the point of the sintered agglomeration of their carbons and a decrease in activity. Therefore, exploring new doping methods to improve the basic framework and surface properties of CNTs seems to be a hot topic in the future.

### 2.2. Mesoporous Carbon

Mesoporous carbon, as a member of the carbon material family, has the common characteristics of a rich pore structure and more functional groups, but also derives the characteristics of a large pore volume, adjustable pore size, disordered mesoporosity, and an ordered porous system [55,56,57]. In addition, the surface chemical modification of mesoporous carbon is also practicable, and the most commonly used modification is the use of nitrogen-containing groups. Nitrogen content is closely related to the catalytic ability, CO_2_ adsorption capacity, and functional group coverage of mesoporous carbon [25,58,59]. Since their discovery, mesoporous materials have been a hot topic in the scientific communities of materials chemistry, physics, and biology, and they also have outstanding properties in the fields of adsorption, separation, and catalysis, so catalysts based on mesoporous carbon are also widely used in FTS [57].

Martin Oschatz et al. synthesised mesoporous templated carbon (CMK-3) with various pore shapes and ordered connections by using mesoporous silica templates, and different surface-functionalised supports were obtained by heating in air, treating in ammonia, and heating in N_2_, respectively. Interestingly, the differences in the porosity, specific surface area, and total pore volume of the treated supports compared with the initial supports were not significant [60,61]. In addition, Na and S additives were also added to the experiment as a comparison, and the experimental results showed that the additives could inhibit methanation, the selectivity of low carbon olefin reached 55%, and the catalytic performance of the N-functionalised support was the best. Unlike the O-functional group, the N-functional group was not heavily encapsulated between the Fe particles and carbon during calcination, so the active sites were in good contact with the syngas, and Na and S enhanced the carbonation of the metal particles, improving the catalytic activity [61]. Similarly, Liu et al. prepared catalysts with different N contents by hard-template-assisted sol–gel polymerisation. In N_2_ physisorption, the highest N_2_ adsorption capacity, specific surface area, and pore volume were observed at melamine/phenol of 1, which was favourable for Fe loading. It should be noted that, after continuing to characterise the catalysts after the reaction, the surface area and the pore volume were decreased, and the nanoparticle particle size increased significantly, indicating that Fe particles sintered with the carbon support and may have accumulated carbon [26]. The experimental results showed that nitrogen-containing groups increased the basicity and improved the reduction and carburisation of Fe, which, in turn, improved the FTS activity, could inhibit methane generation, and could improve the selectivity of low-carbon olefins [26]. Cheng et al. prepared Fe-based catalysts with SiO_2_ and a series of carbon supports, in which CMK-3 consisted of interconnected carbon rods and had smaller pore sizes, exhibiting smaller particle sizes under ethanol solution impregnation than aqueous solution impregnation, with the smaller particles yielding higher CO conversions [21]. On the SiO_2_-loaded catalyst, iron silicate (Fe^3+^), an alkali metal-like electronic property, was formed, resulting in a slightly higher O/P value than the CMK-3 carrier, but the magnetite in the carbon material was able to carbonise faster in the CO activation, thus exhibiting a higher CO conversion and FTY value.

**Figure 4:**
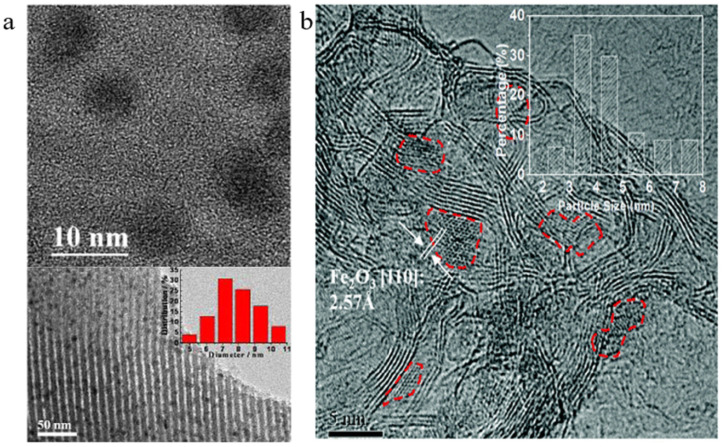
(**a**) TEM image and particle size distribution of Fe-C-8 catalyst [64]. Copyright (2012) American Chemical Society. (**b**) TEM image of Fe/NG_-16.4_, red circles indicate Fe_2_O_3_ nanoparticles, inset indicates Fe_2_O_3_ nanoparticle size distribution [65]. Copyright (2015) ChemComm.

The synthesis of ordered mesoporous carbon containing functionalised particles can currently be carried out either by the hard template method, in which ordered silica is used as a template, or by the soft template method, in which copolymers are co-assembled with a resin precursor in the presence of metal salts, although these methods prepare supports with a degree of deterioration in surface area/porosity and a lack of control over the synthesis process [60,62,63]. Sun et al. reported the synthesis of an ordered mesoporous carbon material with embedded and highly dispersed metal particles using acetylacetone as a chelating agent, and the structural and nanoparticle properties were characterised using SAXS and XRD, etc. The samples had an ordered mesoporous structure, well-crystallised Fe_2_O_3_ particles, and the specific surface area and pore volume were kept stable. Also interesting is that, according to the TEM images, the composites of the Fe_2_O_3_ particles had a unique semi-exposed structure, i.e., one part was exposed in the pore channels and the other part was bound in the carbon framework (as shown in Figure 4a) [64]. The experimental results showed that acetylacetone could promote the growth of nanoparticles and the yield of C_5+_ could reach 68% at a content of 45%, which could still remain active within 100 h. This was also attributed to the limiting effect of the chelating agent as well, and the nanoparticle size changes during the experiments can be neglected, which provides a general synthetic method for subsequent chelation-assisted co-assembly for the preparation of more nanostructures [64].

Its sequential mesoporous structure, unique macropores, and more easily modified surface groups make mesoporous carbon still a hot topic. There are differences in support preparation methods, but their basic characteristics are inseparable from a higher porosity and larger specific surface area. Under some special doping, the transformation of Fe compounds and transfer processes may occur [26,66]. In fact, most of the current mesoporous carbon synthesis still relies on the template method, and we should consider whether the CVD method of CNTs for the growth of carbon atoms can be implemented. Thus, innovative preparation methods and the synergistic effect of multiple additives seem to be the future directions of the research on mesoporous carbon as a support.

### 2.3. Graphene

Graphene, as a new type of carbon structure, is often considered an ideal support material due to its high surface area, unique two-dimensional structure, abundant oxygen-containing groups and defects, excellent thermal conductivity, and ease of modification [67,68,69]. Former common methods for synthesising graphene include electrochemical exfoliation, chemical vapor deposition, and chemical redox [70,71,72]. It should be mentioned that chemical redox seems to be more popular than other methods, not only because of its simplicity of preparation and suitability for large-scale production, but most importantly because graphene (GO) produced by oxidation is able to have an abundance of oxygen-containing functional groups and defects while maintaining a high surface area, which provides more anchor points [72,73,74].

Graphene-doped catalysts with strong metal–support interactions can be used to stabilise small-sized nanoclusters, and their crystal defects are better able to adsorb metals and improve catalyst stability [75,76,77]. For carbon nanotubes without edge defects, edge doping seems unlikely, whereas graphene has serrated edges, which can be doped with heteroatoms such as N and B for the purpose of controlling its electronic structure and chemical properties, also playing a role in activating edge carbon [78,79]. On this basis, Chen et al. prepared Fe/NG catalysts with different N contents by a one-pot solvothermal method and ultrasonic impregnation. HRTEM images showed that the metal particles were well dispersed in the nanosheets (as shown in Figure 4b) and, according to the XRD spectra, N doping was shown to be likely to cause more defects in graphene [65]. It is noteworthy that the selectivity for light olefins could be maintained above 40% for all catalysts obtained under different experimental conditions, whereas usually for iron catalysts under carbon materials without any promoter, the product distribution is very broad and the selectivity for light olefins is generally low [65,80,81]. The excellent conductivity properties of graphene allow this catalyst to have an electronic effect similar to that of alkali metals, which can keep Fe particles in a low-valence state, thus facilitating the FTS reaction.

In addition to conventional heating methods, microwave irradiation (MWI) appears to be becoming a more practical adjunct to reduction methods, using a variety of reducing agents in hot organic solvents for the rapid reduction of exfoliated GO, which allows for rapid and uniform heating and promotes the distribution of metal particles without the need for high temperatures and pressures [82]. Sherif O. Moussa et al. used hydrazine hydrate MWI reduction to prepare K and Mn individually promoted catalysts on graphene oxide carriers. According to Raman spectroscopy, the graphene lattice obtained by this method had more defects, which can be better used as anchors to reduce the agglomeration of Fe particles, and the TEM images also confirmed this, with a better distribution uniformity of metal nanoparticles [39]. The experimental results showed that graphene-loaded Fe-based catalysts had a higher activity and long-chain hydrocarbon selectivity, promoted the formation of carbide active phases, and improved catalyst stability and recyclability, presumably due to the favourable kinetics provided by graphene, as well as a good environment for the nucleation of surface-active nanoparticles.

As one of the more popular carbon materials at present, graphene undoubtedly has great potential. Unlike the high catalytic performance achieved due to the internal nano-channels of CNTs, graphene as a carrier seems to be more inclined to produce CH_4_, but its unique electron density and structure are favourable for the reduction of Fe_2_O_3_ particles. The strategy of improving metal–carrier interactions by designing active sites on the surface of the material and using multiple additives for co-modification has improved the selectivity of graphene-doped catalysts for long-chain hydrocarbons to a greater extent.

### 2.4. Activated Carbon

Activated carbon is one of the commonly used carbon supports in early Fischer–Tropsch synthesis catalysts and, like other carbon supports, has a surface that is easily modified and reduces metal–support interactions, favouring the formation and reduction of the iron carbide active phase [23]. The huge surface area of activated carbon, its better ability to disperse metal particles, and its low cost have led to its extensive research on carbon supports [83].

**Figure 5:**
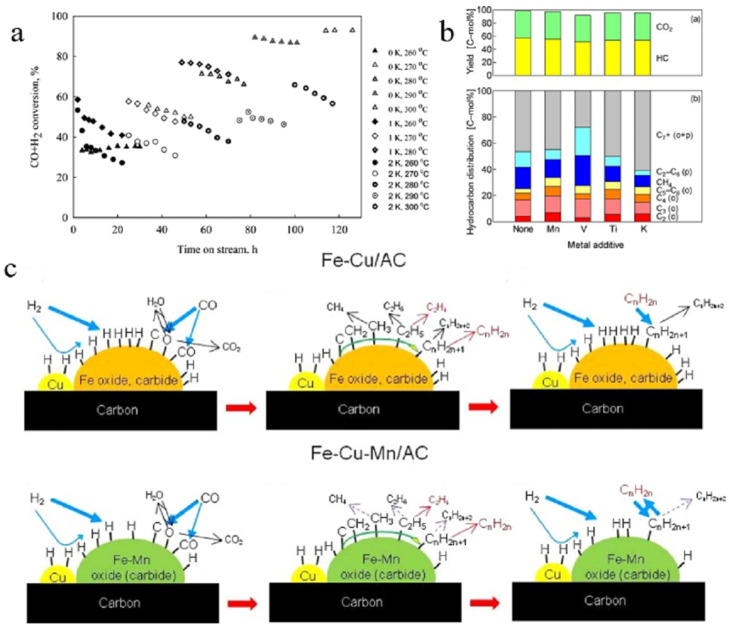
(**a**) Variation in syngas conversion with running time over catalysts [84]. Copyright (2007) American Chemical Society. (**b**) Performance of Fe-Cu/AC with metal addition in low-grade olefin synthesis. (**c**) Surface reaction processes over Fe-Cu/AC and FeCu-Mn/AC catalysts [89]. Copyright (2018) Elsevier.

Most of the current studies have focused on the role of different additives for activated carbon supports in FTS. Ma et al. prepared Fe/K/AC catalysts with varying levels of Fe/K/AC using peat as the source of AC, which was washed, calcined, sieved, and iso-volumetrically impregnated. The results showed that a small amount of potassium (0.9%) increased the activity of the FTS and greatly enhanced the selectivity of the C_5+_ and large-molecular-weight alcohols, while a large amount of potassium (2%) showed a higher activity only initially at low temperatures and the addition of potassium decreased stability (as shown in Figure 5a) [84]. This may have been because potassium inhibits H_2_ adsorption and promotes CO resolution in precipitated iron catalysts [85]. In addition to this, the team calculated the catalyst’s pre-finger factor and activation energy, and showed that potassium additives can significantly reduce both of these metrics [84]. The addition of Mn improved the reactivity and product selectivity by reducing carbon deposition during the reaction and promoting the formation of iron carbide [83,86]. However, it is worth noting that excessive Mn additions can hinder carburisation by forming interactions with Fe, thus affecting the formation of C-C bonds and reducing CO conversion [87]. Therefore, Tian et al. modified an AC-loaded catalyst with different concentrations of KMnO_4_ solution and found that the catalyst crystal size increased with an increase in the KMnO_4_ pretreatment concentration by XRD characterisation. The experimental results showed that too much Mn inhibited the chain growth, and the selectivity of C_5+_ was decreased from 37.4% to 29.7%, which showed an increasing and then decreasing trend [83]. Interestingly, the KMnO_4_-treated AC supports formed a large number of oxygen-containing groups on their surfaces, which facilitated electron transfer and reactivity, increased hydrocarbon yields, and achieved a maximum CO conversion of 85%, according to XPS C1s spectra [83]. Later, the team synthesised nitrogen-doped FeN-MnK-AC catalysts using ferric ammonium citrate as a precursor, and XRD and XPS showed that the nitrogen atoms were not doped into the carbon supports but into the Fe lattice [88]. The results of the comparative experiments indicated that the electronic effect of N and the competitive adsorption of hydrogen seemed to be responsible for the inhibition of the secondary hydrogenation of olefins. Asami et al. investigated the effect of FeCu/AC on olefin formation by the addition of different metals (as shown in Figure 5b), with Mn showing the best effect, and speculated on the surface reaction mechanisms of Fe-Cu/AC and Fe-Cu-Mn/AC catalysts (as shown in Figure 5c), suggesting that the combination of Fe and Mn hindered the adsorption of hydrogen and, thus, reduced the alkyl hydrotreatments [89]. In addition, the experiments also showed that increasing the H_2_/CO value could enhance CO conversion and hydrocarbon yield, while adding CO_2_ could increase HC production without changing its distribution, contributing to the efficient utilisation of CO [89].

### 2.5. Carbon Spheres

Carbon spheres, also known as carbon nanorods and carbon microbeads, are a common name for carbon materials made from carbon sources under oxygen-free and catalyst-free conditions. Currently, the main methods for synthesising carbon spheres are the CVD method and hydrothermal synthesis (HTS). When prepared through the CVD method, the CSs do not need to be purified and have a non-porous surface, which is inert, while preparing carbonaceous spheres by the hydrothermal method enables the metal nanoparticles to be highly dispersed to improve catalyst stability and selectivity. [23]

**Figure 6:**
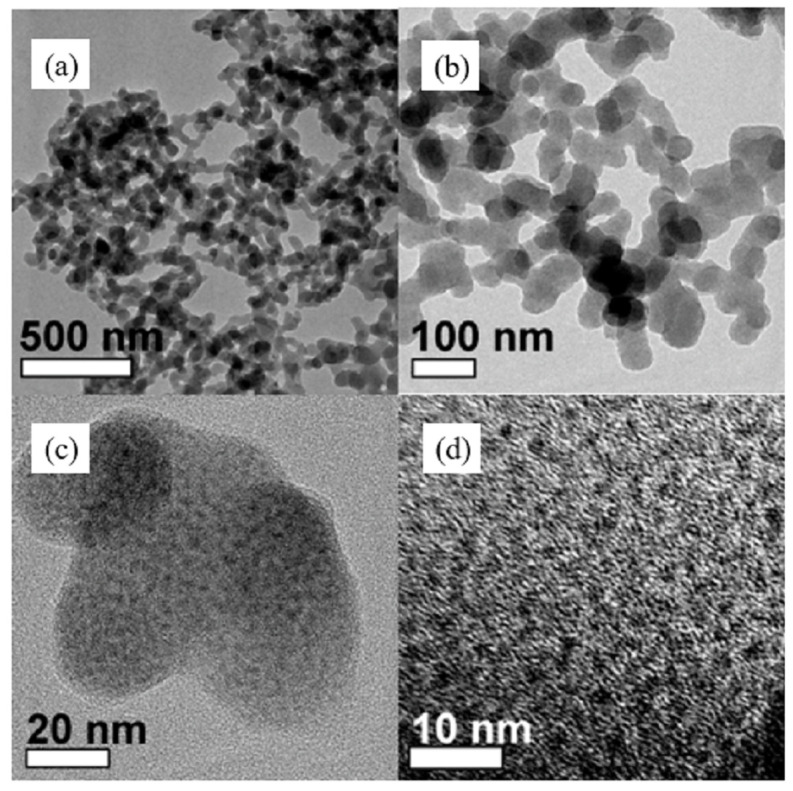
(**a**–**c**) TEM images of Fe_x_O_y_@C spheres at different magnifications and (**d**) HRTEM images of carbon nanorods embedded with Fe_2_O_3_ nanoparticles [92]. Copyright (2009) American Chemical Society.

Most monosaccharides, disaccharides, and polysaccharides, such as glucose and starch, can be hydrothermally treated at about 200 °C to form nano- to micrometre-sized carbon spheres with a surface rich in functional groups [90,91]. Yu et al. prepared Fe_x_O_y_@C spheres by the hydrothermal treatment of iron nitrate and glucose at 80 °C for 24 h. The microstructure was characterised by TEM (as shown in Figure 6) and the atomic ratios of Fe(II) to Fe(III) were estimated by X-ray absorption spectroscopy and fitting curves. The experimental results showed that, due to the catalyst’s promotion of the production of more iron carbide, the olefins and the long-chain hydrocarbons exhibited a high degree of selectivity [92]. Interestingly, replacing glucose with fructose or sucrose or replacing iron nitrate with nickel nitrate or cobalt nitrate could likewise produce similar catalyst structures, and this similar microstructural synthesis approach is universal [92]. Xiong et al. prepared carbon spheres by chemical vapor deposition in a tubular quartz reactor using acetylene as a carbon source and synthesised iron catalysts with different loadings and Cu and K additions using wet impregnation and homogeneous deposition precipitation methods [93]. Various characterisation methods, including XRD, FTIR, TGA, etc., were applied to study the structure and physicochemical properties of the catalysts. Fe loading resulted in a higher BET surface area and pore volume compared to empty loading. The experimental results showed that the KMnO_4_-treated catalysts were comparable to the nitric-acid-treated catalysts with a comparable activity but a higher selectivity of long-chain hydrocarbons and that deposition precipitation gave more dispersed Fe particles with higher space–time yields [93]. In subsequent experiments, Xiong et al. prepared three different nitrogen-doped carbon sphere supports with 5 wt% Fe loading using urea as a precipitant by CVD (vertical and horizontal furnaces) and HTS methods. The experimental results showed that different catalysts had different iron carbide sizes, N contents, and types, although the absence of alkali additives and the lack of a pore structure limited the diffusion of FTS products and inhibited α-olefin resorption and product accumulation, leading to a higher light olefin selectivity [24]. It should be noted that the three catalysts had different Fe particle sizes, as shown by TEM images, which was most likely caused by the different surface areas of the supports and numbers of defective sites, thus affecting the metal particle distribution and FTS activity [24]. It can be seen that carbon carriers are highly sensitive to external conditions, and the surface characteristics of materials obtained by different preparation methods tend to vary, further affecting the physical properties of the metal nanoparticles. For CSs-loaded iron catalysts with the addition of promoters, such as Mn, K, and Cu, the change in catalyst performance and the trend in product selectivity were similar to most carbon-loaded catalysts, so subsequent experiments for CSs supports can produce more work on size and surface chemistry [93].

**Figure 7:**
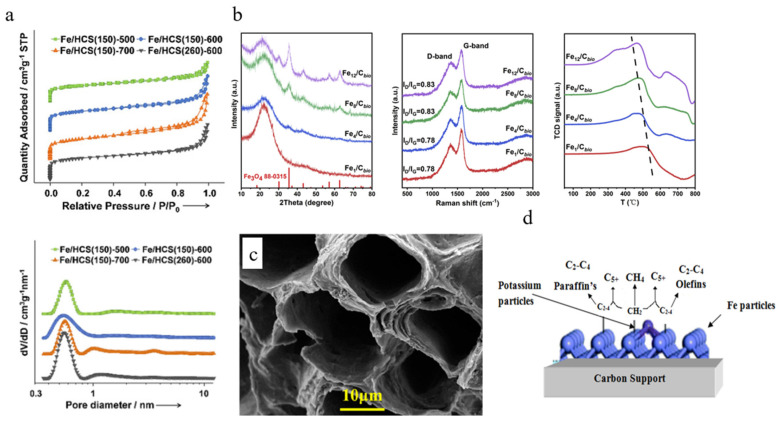
(**a**) N_2_ physisorption isotherms and catalyst pore size distribution [94]. Copyright (2018) Chemcatchem. (**b**) XRD pattern, Raman pattern, and H_2_-TPR pattern of Fe/C_bio_ [95]. Copyright (2021) Elsevier. (**c**) SEM image of the NDPC_bio-2_ support in cross section [96]. Copyright (2022) American Chemical Society. (**d**) Mechanism of a carbon-supported iron catalyst containing potassium as a promoter [97]. Copyright (2022) MDPI.

CNTs have the unique advantage of dispersing the active phase inside and outside the tube, and this structure has also been investigated in hollow carbon spheres. Teng et al. encapsulated a carbon precursor and an iron source on the surfaces of silica spheres and obtained Fe/HCS with Fe_2_C embedded in hollow carbon spheres after pyrolysis and etching. The high temperature promoted the transformation of amorphous carbon to graphitic carbon and the N_2_ physical adsorption characterisation showed that the catalysts had a meso-pore and micro-pore structure (as shown in Figure 7a) [94]. The experimental results showed that, although the initial catalyst activity decreased with an increasing pyrolysis temperature, the deactivation was slowed down, and increasing the inner diameter of the carbon spheres promoted the dispersion of Fe on the carbon substrate, increasing the catalyst stability and improving the CH_4_ selectivity.

### 2.6. Biomass Derived

So far, various catalysts based on mesoporous carbon, carbon nanotubes, graphene, etc., have emerged [51,66,98]. However, the lack of understanding of the metal–support interactions of carbon materials, including problems such as the sintering of catalyst particles, wax accumulation, and carbon methanation, has limited their large-scale industrial application [99,100]. Therefore, exploring novel carbon materials is crucial to improve the reaction performance of CO_2_-FTS and extend the catalyst lifetime. Biomass carbon-based supports have the advantages of a high pore volume, large specific surface area, and wide range of applications, as well as a wide distribution, easy availability of raw materials, and high renewability; in addition, their abundant surface functional groups make it convenient to regulate the catalyst metal–support interactions and promote the formation of iron carbide, which is the active substance [101,102].

Based on the excellent performance of biomass-based carbon supports, Bai et al. prepared a series of Fe/C catalysts with different Fe loadings produced by a simple impregnation method. By comparing the catalysts with blank supports, AC supports, XRD patterns, Raman patterns, and H_2_-TPR patterns (as shown in Figure 7b), excellent metal–support interactions characterised by using sugarcane as the carbon substrate were revealed, as well as an optimal Fe loading of 4 wt%. In addition, SEM analysis showed that the biochar support had an extremely high 3D porous layered structure and Fe could be sufficiently and uniformly dispersed on the surface [95]. The active phase of the FTS reaction that is now generally accepted is iron carbide, formed by the carburisation of iron oxides [103]. At the conventional reaction temperature of 543 K, many reports consider χ-Fe_5_C_2_ as the main active phase of the reaction [104,105]. However, recent studies have shown that ε-Fe_2_C is more active than χ-Fe_5_C_2_ in LTFTS and prepared ε-Fe_2_C nanocrystals, with a high stability under high-temperature conditions [106,107]. Therefore, the high activity exhibited by the above sugarcane support could provide more functional groups to interact with the metal particles, reduce the agglomeration of the metal particles, improve the dispersion of Fe, and favour the generation of ε-Fe_2_C, in addition to the rich three-dimensional layered pore structure. The results of this experiment showed that the catalyst had a better selectivity for methane and short-chain hydrocarbons, but the long-chain hydrocarbon products were generally lower than those of other common catalysts [95]. At a low Fe loading of 4 wt%, the catalytic activity was much higher than the reported level of AC carriers, thus it seems that, compared to common carbon materials, a large number of functional groups are present on the surface of natural biomass, and unlike artificially modified surface properties, this natural functional group is more stable, with the catalysts still able to remain stable at TOS = 150 h.

In order to improve the product distribution characteristics, Bai et al. prepared a series of nitrogen-doped bioporous charcoal with different nitrogen contents by adding urea, and the results showed that the nitrogen content had a significant effect on the selectivity of C_5+_ and showed a parabolic trend, which reached the peak at a mass ratio of urea to K_2_CO_3_ of 2 [96]. Iron nanoparticle size has a strong influence on catalytic activity and stability, with smaller particle sizes exhibiting a higher CO adsorption strength and a stronger selectivity for long-chain hydrocarbons [108]. On this basis, Bai et al. obtained different contents of nitrogen structures by varying the nitrogen source reagents. SEM images showed that all the nitrogen-doped biochars had a high BET surface area and abundant pore structures (as shown in Figure 7c), among which pyrrolic N not only preferentially promoted the growth of iron nanoparticles, but also provided more anchors, improved the high dispersion of iron, and reduced the crystallinity of the catalysts [96]. In order to study the effects of temperature and the promoter on the distribution of liquid hydrocarbons, Muhammad Amin et al. investigated the conversion of syngas with K as a promoter at low (200 °C) and high (350 °C) temperatures using Lantana Camara as a biomass carbon support. The results of the experiments showed that the inclusion of K and high temperatures contributed to the formation of iron carbide, which was able to better improve the gasoline (72%) and diesel selectivity [97]. Of interest, the team demonstrated through SEM images that the experimental biomass-activated carbon was internally covered with pores and that its chambers were upright, which prolonged the contact time between C and Fe particles [97]. In fact, the carbon material contributed to the carbide formation of Fe, which promoted the production of branched alkanes to achieve carbon chain extension, whereas K merely promoted and did not change this mechanism (as shown in Figure 7d). Overall, although the sources of biomass were different, when using them as supports, all of them exhibited an extremely high surface area and rich pore structure, which was conducive to improving the dispersion of Fe nanoparticles.

Carbon-based supports derived from biomass, including carbon nanotubes and carbon nanorods, are currently widely used in multiphase catalysis, and the future preparation of ‘green’ catalysts will require the expansion of biomass sources, as well as the optimisation of synthesis methods to provide sustainable processes [109,110].

### 2.7. Derivation of MOFs

Carbon-containing supports, including carbon nanospheres, activated carbon, and carbon nanotubes, have advantages in terms of a higher specific surface area and a porous structure, but supports made by the carbonisation of organic precursors have a greater impact on the distribution of the active phase at high-temperature conditions, as well as at high loadings [111]. Therefore, inorganic supports have been extensively studied in recent years, with metal–organic frameworks (MOFs) being repeatedly investigated. In addition to possessing universal porous material properties, MOFs are characterized by a moderate to high chemical and thermal stability, decoratable pores with different functional groups, and organic–inorganic hybrid properties [112]. In addition, MOF materials are flexible in practical applications and can be integrated with functional materials (metal nanoparticles, silica, and polymers, etc.) to improve their catalytic activity, stability, and give full play to their catalytic potential [113]. Compared with conventional catalyst particle agglomeration, oxidation, and deactivation, Fe@C nanoparticles prepared with MOFs can be effectively confined in the carbon matrix and the precursor can be properly adjusted in terms of the catalyst size, Fe loading, and carbonised iron phase by different carbonisation methods [111].

**Figure 8:**
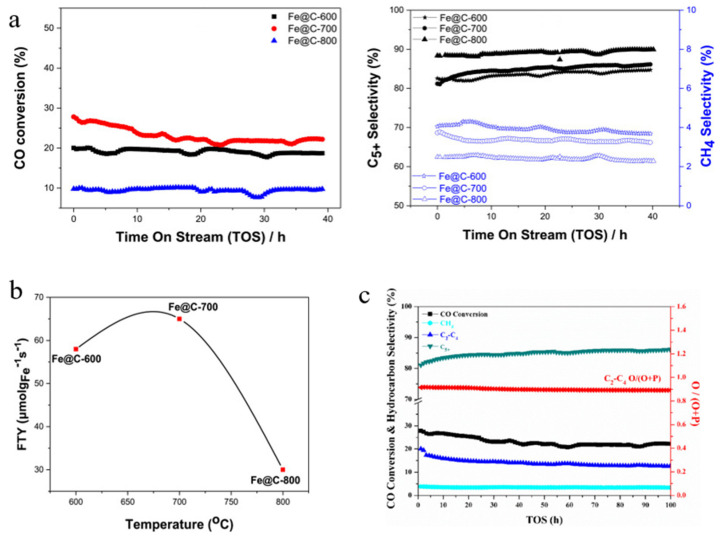
(**a**) CO conversion and selectivity of Fe@C catalyst with time, (**b**) time yield at different temperatures, and (**c**) activity and selectivity of Fe@C-700 catalysts [115]. Copyright (2022) Elsevier.

Wezendonk et al. synthesised Fe@C-500 and Fe@C-600 at different pyrolysis temperatures via MOF-mediated synthesis. Situ Mössbauer absorption spectroscopy showed that both sets of catalysts were transformed to χ-Fe_5_C_2_ by carburisation under HTFT and to ε’-Fe_2.2_C by reduction under LTFT with the same tendency. At the same time, there was a significant difference in the selectivity for the products, which was related to the method of activation, namely the iron carbide phase, and both reduced and carburised catalysts showed a high selectivity for C_5+_ [31]. Previously, the team prepared catalysts by pyrolysis at different temperatures using Fe-BTC as a precursor, showing that the type of iron carbide formed, the activation behaviour, and the amount of partitioning depended on the pyrolysis temperature and that the methane selectivity tended to decrease as the temperature increased, i.e., the Fe loading and activation phase could be controlled by adjusting the pyrolysis temperature [114]. Similarly, Mehar U. Nisa et al. used Fe-MIL-88B as a precursor and after pyrolysis at different temperatures and reductions under hydrogen conditions, which, according to PXRD characterisation, yielded predominantly Fe_3_C and Fe_5_C_2_ at 600 °C and 700 °C, whereas Fe_3_C was transformed to Fe_7_C_3_ and a small amount of Fe at 800 °C [115]. The literature has shown that a smaller support pore size is more likely to prolong the contact of the reaction intermediate with the iron catalyst, thus favouring the generation of long-chain hydrocarbons [116]. Therefore, the catalysts pyrolyzed at 800 °C in the above experiments showed a better selectivity for C_5+_ (as shown in Figure 8a), and it is noteworthy that the catalysts obtained by pyrolysis at 700 °C had the highest FTY and CO conversions (as shown in Figure 8b,c), most likely due to the higher activity of Fe_5_C_2_ compared to Fe_7_C_3_ [115,117]. In order to optimise the Fe@C catalysts, Tim A. Wezendonk et al. selected some commercially available Fe-MOFs (MIL-68, MIL-88A, MIL-100, MIL-127, etc.) to investigate the activity and product selectivity of MOFs in HT-FTS, and it was demonstrated by SEM that the morphology of the MOFs did not change after pyrolysis, but the crystallinity and Fe nanoparticles varied in size, with the results indicating that the catalytic activity of Fe@C was closely related to the porosity of the precursor, the crystallinity after pyrolysis, and the loading of Fe [118]. Cho et al. prepared FeC(x,y) catalysts (x is the time and y is the pyrolysis temperature) using MIL-100 (Fe) as a precursor, in which the main crystalline phase was Fe nanoparticles and the particle size increased with temperature and time [119]. The experimental results showed that FeC(4,700) had a higher catalytic activity, which was mainly attributed to its smaller and stable FeO_x_ particles, the easy formation of the χ-Fe_5_C_2_ phase on the surface, thin carbon layer, large surface area, and small aggregation. In conclusion, obtaining the optimal catalyst for the reaction requires a comprehensive consideration of the precursor structure, the influence of heteroatoms, and the reaction conditions.

In conclusion, although there are differences in precursors or preparation methods, MOF materials all seem to improve the dispersion of Fe particles confined within the porous carbon matrix, and close contact between Fe and C promotes the formation of the active phase of iron carbide [120]. MOF templating methods are still in the early stages, and this emerging porous material as a support has unique limiting effects and spatial control. In the future, the selection of highly stable MOF materials and the enhancement of their applicability through later modification are worthwhile for continued development [111,121]. Table 1 shows the structural parameters of the CO hydrogenation catalysts and Table 2 summarises the catalytic performance of the different catalysts.

## 3. CO_2_ Hydrogenation Reaction

The hydrogenation of CO_2_ to obtain hydrocarbon products is mainly achieved through two methods, namely the methanol intermediate route (as shown in chemical Equations (1)–(4)) and the CO intermediate route (as shown in chemical Equations (5) and (6)). Relatively speaking, the CO intermediate route is more economical, with fewer chemical process steps and a lower energy consumption [122,123].

CO_2_ + 3H_2_ → CH_3_OH + H_2_O (1)

2CH_3_OH → CH_3_OCH_3_ + H_2_O (2)

nCH_3_OH + H_2_ → CH_3_(CH_2_)_n−2_CH_3_ + nH_2_O (3)

nCH_3_OH → CH_2_ = CH(CH_2_)_n−3_CH_3_ + nH_2_O (4)

CO_2_ + H_2_ → CO + H_2_O (5)

nCO + (2n + 1)H_2_ → C_n_H_2n+2_ + nH_2_O (6)

**Figure 9:**
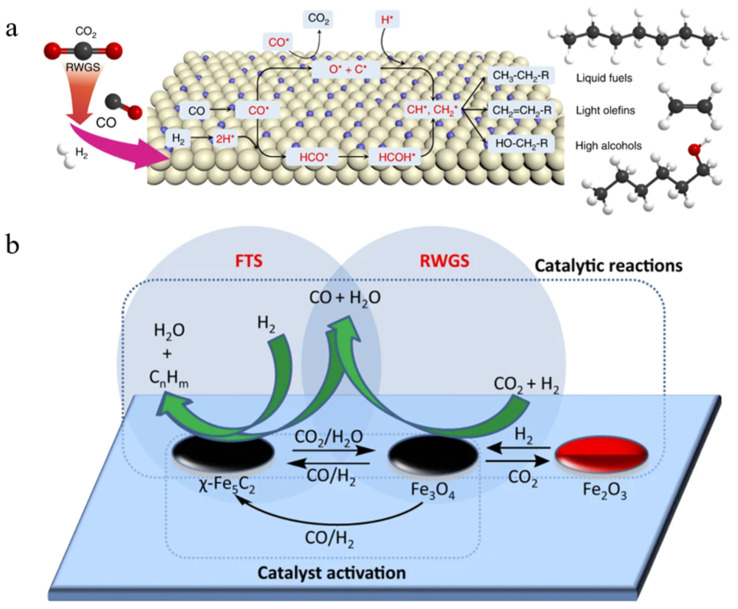
(**a**) Scheme of CO_2_-modified FTS-based catalytic mechanism [122]. Copyright (2019) Springer Nature. (**b**) The CO_2_ hydrogenation to jet fuel range hydrocarbons process through a tandem mechanism in which the reverse water–gas shift reaction (RWGS) and Fischer–Tropsch synthesis (FTS) reaction are catalysed by Fe_3_O_4_ and χ-Fe_5_C_2_, respectively [123]. Copyright (2020) Springer Nature.

It is generally believed that the mechanism of producing C_2+_ through the CO intermediate pathway mainly occurs through a two-step method, namely RWGS and FTS, also known as CO_2_-FTS (as shown in Figure 9a,b) [122,123]. Therefore, it is very important to find a bifunctional catalyst and explore the delicate balance between the two-step reactions. Currently, Co- and Fe-based catalysts are the most popular, however, Co-based catalysts are less commonly used in CO_2_-FTS alone due to their inactivity for the RWGS reaction and high CH_4_ yields due to their high hydrogenation capacity for CO [124]. Comparatively, Fe-based catalysts are reactive for both RWGS and FTS and have better C-C coupling during CO_2_ hydrogenation, which is also favourable for the production of hydrocarbon products (as shown in Figure 9b) [123,124,125]. Interestingly, CO_2_ hydrogenation performance is closely related to the ratio of Fe_5_C_2_/Fe_3_O_4_, with higher ones failing to produce sufficient CO* concentrations and lower ones being unfavourable for FTS reactions [126]. For this tandem reaction, how to regulate the active phase ratio and synergism seems to be the focus of subsequent catalyst preparation. In addition to this, the CO_2_-FTS reaction has a high structural sensitivity, and the size of the metal nanoparticles has a high impact on the reaction performance. Several studies have shown that RWGS and methanation reactions are more sensitive to particle sizes of 6.1–12.9 nm, while FTS favours sizes of 2.5–9.8 nm [11]. Fe nanoparticles were in the range of 4.7–10.3 nm and the particle size was positively correlated with the selection of carburisation and long-chain hydrocarbons [127]. Thus, carbon supports seem to have unique advantages in CO_2_-FTS. Compared with metal oxide supports, carbon supports can reduce the nanoparticle size as a whole and exhibit a highly structured morphology with a multistage porous structure, which can effectively inhibit the agglomeration of the active phase [125].

### 3.1. Carbon Nanotubes

For CNTs, there can be differences in conversion and selectivity between iron nanoparticles on the outer surface and within the pores, and some powdered CNTs may have a higher pressure drop and agglomeration during preparation. To alleviate some of these limitations, Minett et al. grew CNT arrays on cordierite monoliths by aerosol chemical vapor deposition (CVD). In comparison to the powdered form, they found that the monolithic catalysts were more able to withstand high pressures, and that the CNTs prepared by this route were able to control oxidative activation, resulting in similar conversions to the powdered catalysts, with higher reaction rates at high pressures [128]. Discharge plasma sintering (SPS) allows for higher heating rates to be achieved by pulsed currents of the sample at high pressures, increasing the density of the sample as the sintering pressure and temperature are increased, resulting in the formation of a 3D framework structure, which is commonly used for the preparation of bulk CNT materials [129,130]. In fact, the core–shell structure of the CNT backbone structure with active nanoparticles enhances the stability of the SPS catalyst against sintering, and close contact between Fe and the carbon support promotes the rapid generation of iron carbide [130]. Chernyak et al. prepared CNTs by CVD followed by continued treatment with nitric acid to establish anchoring sites for metal nanoparticles, which were then loaded with Fe by impregnation and SPS techniques at different temperatures. XRD and TEM analyses demonstrated that the SPS treatments reduced and carburised the iron oxide particles, but there was a difference in the physical phases and particle sizes of the sintered particles at different temperatures [131]. The results of this experiment showed that the SPS treatment method can embed metal nanoparticles into dense CNT frameworks with the advantages of strong metal–support interactions, carbon support facilitation, and supercritical conditions, resulting in a high CO_2_ hydrogenation activity and selectivity [131]. However, at present, it seems that the increased equipment cost, high temperature, and high-pressure harsh processing conditions that make Fe nanoparticles sintered and agglomerated seem to limit the scaling up of SPS technology for use on carbon carriers.

**Figure 10:**
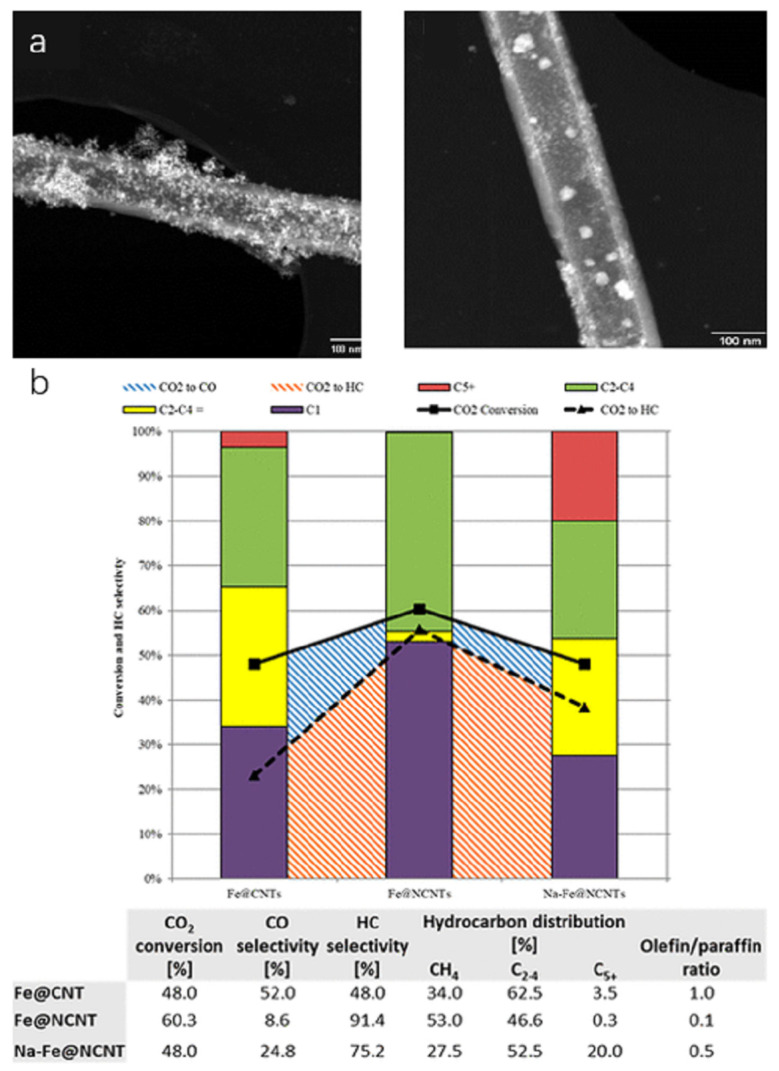
(**a**) STEM image of Fe/OCNT and Fe/NCNT [51]. Copyright (2014) Elsevier. (**b**) Effect of nitrogen doping on the reactivity of RWGS/FTS combination [132]. Copyright (2019) American Chemical Society.

Chew et al. first treated multi-walled carbon nanotubes with nitric acid vapour to obtain oxygen-containing functional group support OCNTs and then treated them in ammonia to obtain nitrogen-containing group support NCNTs. According to STEM, Fe/NCNT iron oxide nanoparticles were mostly inside the CNT channels, while Fe/OCNTs were mostly on the outer wall (as shown in Figure 10a). TPR showed that the Fe/NCNTs had a much lower reduction temperature, and were easier to reduce [51]. The experimental results showed that both catalysts had a high olefin selectivity and chain growth probability, indicating that a large number of Fe carbonation products were generated during the catalytic process, and interestingly, the distribution of Fe nanoparticles in the CNT mentioned above seemed to have a lesser impact on the catalytic performance [51]. Fe/CNT and Fe/NCNT were prepared by Williamson et al. Although nitrogen doping can provide electron-rich anchors to improve dispersion and particle size, the particle sizes from this experiment were within the error of each other and can be considered similar [132]. The experimental results showed that the Fe catalyst could enhance the reducibility by the electrons provided by doping with nitrogen and significantly increased the conversion of CO_2_ and CO, however, nitrogen doping may be more favourable to improve methane selectivity than the production of long-chain hydrocarbons (as shown in Figure 10b).

In the CO_2_ hydrogenation reaction, pre-reduction is an important part, and both nitrogen doping to provide electrons and SPS technology promote catalyst reduction to some extent. Unlike the collapse phenomenon of MOFs materials, for CNTs carriers, SPS technology can embed metal particles and cover some surfaces of the particles with carbon shells, similar to the core–shell structure, so as to realize reduction during the SPS treatment. This seems to provide an idea for innovation in subsequent preparation methods.

### 3.2. Mesoporous Carbon

**Figure 11:**
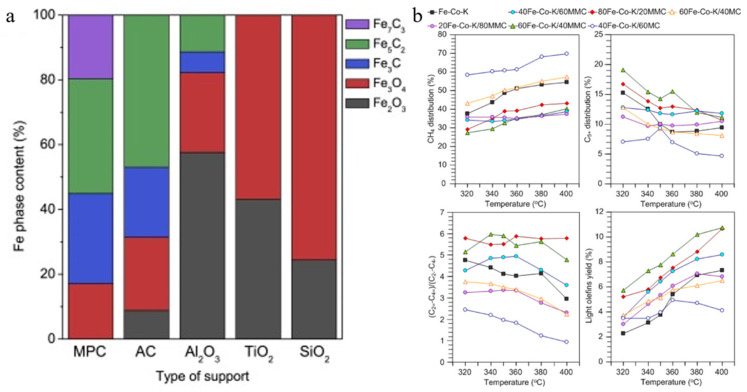
(**a**) Ratio of Fe oxides to Fe carbides calculated from XRD [66]. Copyright (2020) Elsevier. (**b**) Variation in products with reaction temperature [134]. Copyright (2022) Elsevier.

Sun-Mi Hwang et al. prepared mesoporous carbon (MPC) and catalysts by nanocasting and ultrasound-assisted melt infiltration using mesoporous silica spheres (MPSSs) as hard templates. Using various characterisation techniques, the MPC had a highly porous structure with an average pore size of 6.9 nm, and the unique macroporous structure was conducive to the formation of small metal particles. The weaker metal–support interaction also significantly reduced the reduction temperature of Fe_3_O_4_ [66]. The relevant literature suggests that the environment of Fe ions in the oxide precursor controls the Fe_x_C_y_/Fe_x_O_y_ ratio and influences the activated catalyst surface atoms, which alters the CO_2_ selectivity and the yield of liquid hydrocarbons [133]. Therefore, this explains the higher catalytic activity and C_5+_ selectivity of FeK/MPC compared to other support catalysts in the above experiments, which had multiple Fe-C compounds (as shown in Figure 11a) and a mesoporous structure, which was conducive to the improvement of the hydrocarbon mass transfer rate [66]. The large particle size and pore size enabled the Al_2_O_3_ carrier to exhibit a high selectivity for light olefins, while the strong interaction between Fe and SiO_2_ resulted in the absence of Fe carbons in the catalysts and a lower catalytic performance. In contrast, the small particle size and abundant carbons on the MPC carriers gave them unique activities. Witoon et al. investigated catalysts under bimodal microporous mesoporous carbon (MMC) and unimodal microporous carbon (MC) as supports; MC had a higher dispersion of metal oxides, but also resulted in low contact interfaces between the particles, whereas the aggregation of the metal particles in MMC provided greater contact and promoted olefinic selection (as shown in Figure 11b) [134].

### 3.3. Graphene

**Figure 12:**
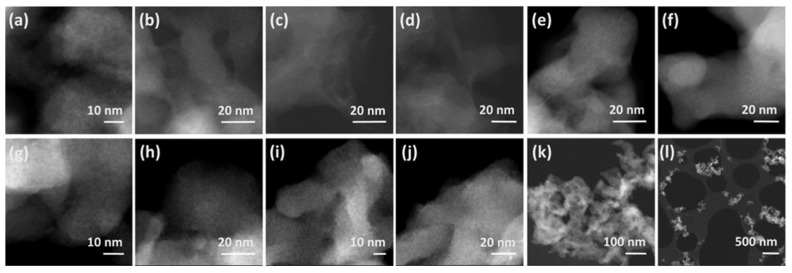
DF-TEM images of samples 1–5 ((**a**,**b**,**k**,**l**): sample 1; (**c**,**d**): sample 2; (**e**,**f**): sample 3; (**g**,**h**): sample 4; and (**i**,**j**): sample 5) [135]. Copyright (2021) American Chemical Society.

**Figure 13:**
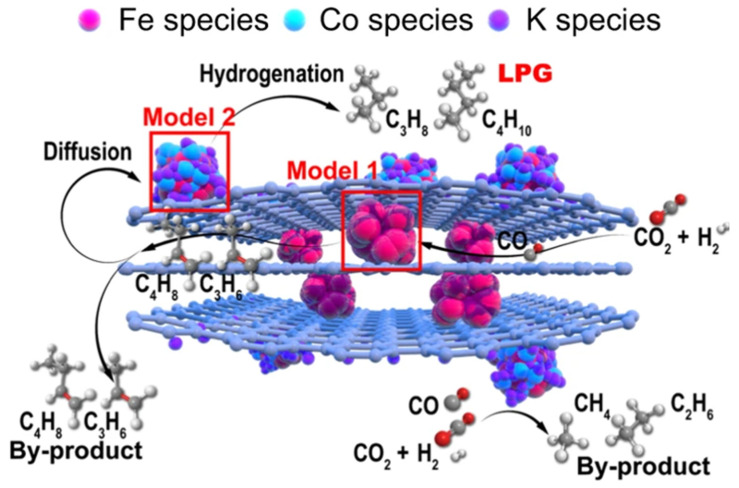
Reaction path diagram. Fe species, red balls; Co species, light blue balls; K species, purple balls [136]. Copyright (2024) Springer Nature.

In order to determine the catalytic activity of clusters loaded with metals on graphitic carbon for carbon dioxide hydrogenation, Peng et al. prepared nitrogen-doped Co, Fe, and Co-Fe catalysts with different contents by supercritical drying and pyrolysis. FESEM showed that the samples were in the form of multi-hollow fibres, and TEM images indicated that the low-metal samples were mostly clusters (as shown in Figure 12), while the high-metal samples were mostly nanoparticles and were distributed at the edges and central ridges of the supports [135]. Although the metal content in some samples was lower, the cluster series was more active and the highest CO selectivity reached 98%, demonstrating the higher catalytic performance of N-doped graphene-loaded sub-nanometre-scale Co-Fe clusters, although, unfortunately, the stability and selectivity of such cluster-aggregated particles decreased significantly beyond the tested duration, and their service life was shorter [135]. Liang et al. reported a graphene fence engineering approach to modulate multiple active sites in Fe-Co catalysts, and the mechanism was mainly cluster connection and penetration of metal particles (as shown in Figure 13). A reduction in graphene layer spacing and cross-linking during hydrothermal treatment led to the transformation of a 2D layered structure into a 3D structure, with dispersed spatial distributions of Fe and Co in the segregated inner and surface layers, which reduced deactivation due to metal agglomeration [136]. The experimental results showed that GO-Fe/K-Co had a 43.6% LPG (propane and butane) selectivity, which was mainly due to the synergistic action of the separated Fe-Co bis-active sites, which promoted the RWGS and FTS reactions from the internal Fe-active phase and generated olefin diffusion to the external bimetallic active sites with a strong H_2_ adsorption capacity, which could simultaneously satisfy the growth of the carbon chain and the secondary hydrogenation of olefin.

In addition to the common method of preparing 2D nanosheet graphene, there is a need to explore how to change the structure to solve the drawback of its inability to restrict the aggregation of active ingredients. Wang et al. synthesised a novel 3D honeycomb structure graphene (HSG) through a simple reaction of Li_2_O with CO, which can be used as an excellent support with a high porosity and high electrical conductivity [137]. Using this method, Wu et al. prepared Fe-K/HSG with different contents, whose basic physical properties did not differ much from those of conventional 2D graphene according to characterisation techniques such as XRD, but the HSG had a mesoporous structure and a porous framework that could accommodate more magnetite. The experimental results also demonstrated that the catalysts with a content of 1.5% K had a very high FTY and light olefin selectivity [95].

### 3.4. Activated Carbon

**Figure 14:**
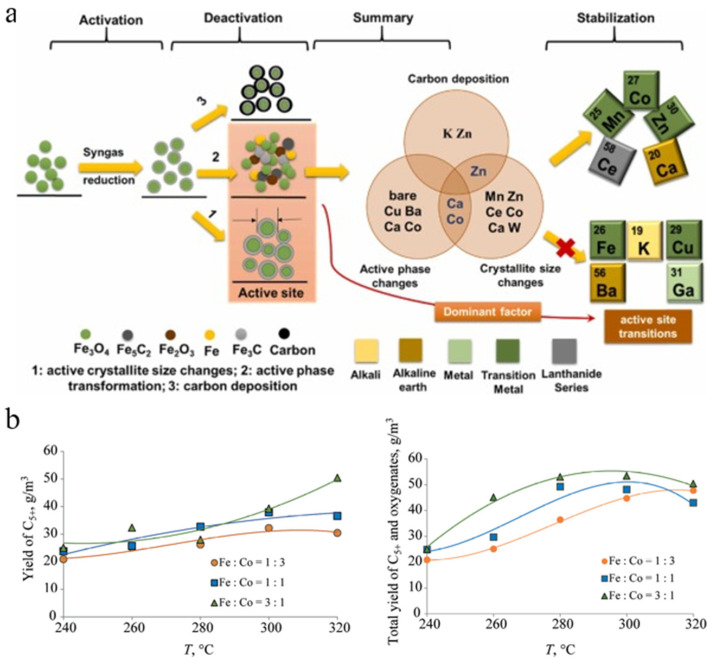
(**a**) Schematic diagram of different promoter inactivation [139]. Copyright (2023) Elsevier. (**b**) C_5+_ yield and total oxygenated compound yield of catalysts with different active components as a function of reaction temperature [140]. Copyright (2023) Springer Nature.

Felgueiras et al. compared the effects of different supports (AC, CNT, and Al_2_O_3_) on CO_2_ conversion and LCOH selectivity, independently of the loaded metal. The AC support showed a higher specific surface area value, and unlike the agglomeration of the metal particles in the CNT, the AC was more homogeneous with the distribution of Al_2_O_3_ [138]. CNT and Al_2_O_3_ are essentially free of micropores and exhibit mesoporous structures, reducing diffusion limitations. Although the AC support exhibited the smallest metal particle size values, the experimental results showed that smaller particle sizes were not favourable for the catalytic results and preferred CH_4_ production. Chen et al. investigated the effects of a series of metal additives on the deactivation, selectivity, and activity of Fe/AC catalysts, and according to the characterisation results, the catalyst deactivation mainly depended on the active site transformation and coke deposition, with the effects exhibited by different additives varying (as shown in Figure 14a) [139]. Although the oxidation of Fe carbide was the main causative factor for deactivation, the additive-induced increase in the microcrystalline size of the active phase of Fe_5_C_2_ compensated for this deactivation by exposing more active sites. The experimental results showed that the modification of Zn protected the active sites better compared to the other additives and that this stabilising activation function also promoted an excellent olefinic selectivity.

### 3.5. Biomass Derivation

Svidersky et al. prepared Fe, Co, and Fe-Co catalysts with different molar ratios using biochar as a support, and the experimental results showed that the highest point C_5+_ yield was achieved when Fe/Co was 3, while the temperature values at which the maximum growth interval was located seemed to be somewhat different when the ratio was varied (as shown in Figure 14b), and that the interactions of Fe and Co prevented methanation site production [140]. In fact, the use of biochar as a support facilitated the synergistic action of the bimetallic active centres, and microcrystals of the active phase were generated from the Fe-Co alloy co-clusters, which inhibited the formation of the massive mixed oxide phase and prevented the agglomeration of the active components.

**Figure 15:**
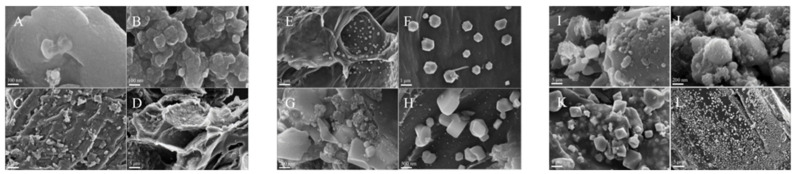
SEM images of dry LC (**A**,**C**), carbonised LC (**B**,**D**), Fe/LC-0.2 carbonised at 700 °C (**E**,**F**), 650 °C (**G**), and 800 °C (**H**), Fe/LC catalysts with Fe/LC mass ratios of 0.08 (**I**,**J**), 0.4 (**K**), and 1.0 (**L**) [102]. Copyright (2024) The Royal Society of Chemistry.

A large body of literature demonstrates that K acts as a promoter of Fe-based catalysts during FTS to improve catalytic activity and inhibit the methanation reaction, increasing the carburisation rate of Fe [141,142]. In addition to the external K source, the use of natural K elements from biomass is also an excellent choice. Zhu et al. designed a simple synthesis idea by directly using waste lychee shell (LC) as a natural carbon support. The morphology of LC before and after carbonisation was observed by the SEM technique, which was transformed from a lamellar to laminar structure, exposing more active sites (as shown in Figure 15). The different carbonisation temperatures led to different porous structures and roughness and affected the dispersion of the active phase and the agglomeration phenomenon of Fe_3_O_4_. The experimental results showed that the selectivity of LCs could reach 47% at a carbonation temperature of 700 °C and an Fe/LC loading ratio of 0.2, and the catalysts could still maintain excellent activity within 60 h. The results of the carbonation of LCs are summarized in the following sections [102]. In addition to this, the team also verified the effect of the promoter by removing the K source from the catalyst, and the results showed that either the added K source or the natural K in the LC could inhibit the secondary hydrogenation of olefins and improve the selectivity of light olefins [102].

### 3.6. MOFs Derivation

**Figure 16:**
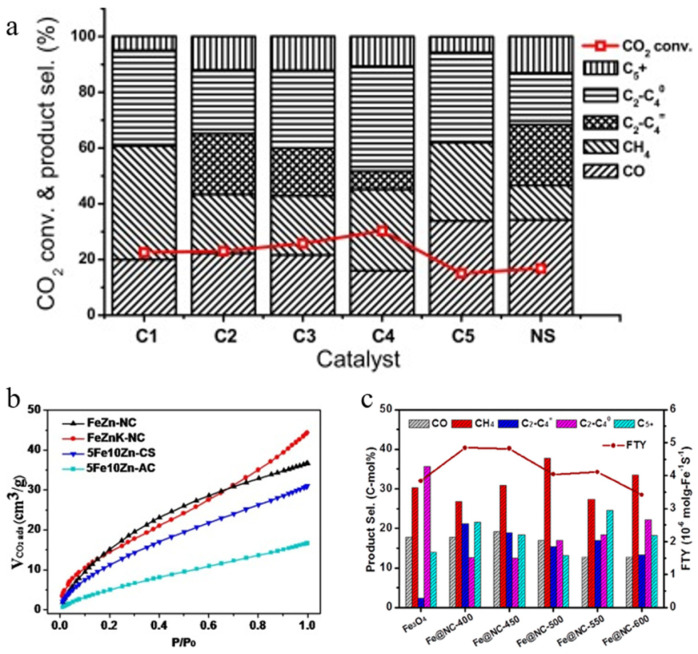
(**a**) Effect of different catalysts on CO_2_ conversion and product selectivity [143]. Copyright (2016) Elsevier. (**b**) CO_2_ adsorption performance of catalysts [151]. Copyright (2016) Elsevier. (**c**) FTY and product distribution of Fe_3_O_4_ and Fe@NC catalysts [152]. Copyright (2019) American Chemical Society.

Hu et al. prepared MIL-53(Al) with different morphologies and ZIF-8 with different sizes, using γ-Al_2_O_3_ as the support for comparison, and the results showed that the hydrogenation reaction was more pronounced due to the acidic nature of MIL-53(Al) and Al_2_O_3_, resulting in a very high alkane selectivity and significantly higher selectivity for low-chain hydrocarbons than C_5+_ (as shown in Figure 16a) [143]. The literature shows that ZIF-8 has more hydrogen adsorption sites and exhibits a good hydrogen storage capacity [144,145]. Thus, the olefin selectivity exhibited in the experiments decreased with an increasing ZIF-8 particle size [143]. In addition, it was shown by X-ray diffraction that the framework of the reacted MOF material was basically unchanged and the crystallinity was slightly reduced, which proved that the material had a better hydrothermal stability and was suitable for catalytic reactions at high temperatures and pressures [143]. Compared to other metal additives (Cu, Mo, Mg, Zn, etc.), only K and Na seemed to improve olefin selectivity. Adrian Ramirez’s experimental team prepared K-promoted catalysts by an MOF-mediated method, and TEM showed that Fe nanoparticles were bound in the carbon pores and Fe_5_C_2_ and Fe_7_C_3_ were observed in XRD. The experimental results showed that, in FTS, Fe_5_C_2_ seemed to be the more preferred active site in FTS, which favoured olefin resorption and led to a higher olefin selectivity [146].

It was shown that nitrogen doping could effectively improve the CO_2_ trapping capacity [147,148]. Moreover, the high electron affinity and adsorption capacity of nitrogen affect the distribution of metal particles, which can stabilise Fe in the low-valence state, thus improving the selectivity of olefins [149,150]. Liu et al. prepared Zn-NC, FeZn-NC, and FeZnK-NC by pyrolysis using ZIF-8 as a precursor, while CS and AC carbon loadings were used as comparisons. XRD showed that FeZn-NC retained the structure of ZIF-8 and had a much higher BEM surface area than the remaining two groups of catalysts, and TEM images showed that FeZn-NC particles were encapsulated by carbon layers with many cavities. The CO_2_ adsorption capacity was also investigated, and the results showed that the nitrogen-doped catalyst had a higher adsorption capacity (as shown in Figure 16b) and the catalyst had a stable selectivity for long-chain hydrocarbons and light olefins, with C_2_~C_4_ as the dominant ones [151]. This was mainly due to the fact that Zn acts as a structural aid to enhance CO adsorption on the Fe surface and that N can act as an electron donor to enhance the adsorption–dissociation of CO_x_. In order to investigate the specific catalytic properties of nitrogen doping, Liu et al. firstly prepared Fe_3_O_4_ nanoparticles by the solvothermal method and mixed them with ZIF-8 to obtain Fe_3_O_4_@ZIF-8. The catalysts were obtained in a nitrogen atmosphere at different temperatures, which were characterised by XRD to show the presence of different iron species in the catalysts with different treatments and the decrease of BEM surface area with an increase in temperature [152]. The experimental results showed that the introduction of nitrogen resulted in a 24-fold increase in O/P and a much higher reaction rate and light olefin selectivity compared to the benchmark Fe_3_O_4_ catalyst (as shown in Figure 16c) [152]. Xu et al. investigated the effects of different pyrolysis temperatures on NH_2_-MIL-88 B-derived Fe-based catalysts. Two-stage pyrolysis mitigated the surface area reduction and structural collapse induced by the temperature increase, promoting the formation of Fe_3_O_4_-Fe_3_C structures with smaller particle sizes and more concentrated distributions [153]. In addition, the organic ligand NH_2_-BDC provided a nitrogen source for the reaction, and lower temperature pyrolysis exhibited higher CO_2_ conversion as the pyrolysis temperature was increased, with lower pyridine nitrogen and higher graphite nitrogen, and pyridine nitrogen could increase CO_2_ hydrogenation activity. Table 3 shows the structural parameters of the CO_2_ hydrogenation catalysts and Table 4 summarises the catalytic performance of the different catalysts.

## 4. Summary and Challenges

The hydrogenation of CO_x_ to synthesise high-value-added hydrocarbons is an important way to solve the energy and environmental crisis. Among many catalysts, Fe-based catalysts are widely used due to their ability to catalyse both RWGS and FTS reactions, although the resulting wide distribution of catalytic products and poor catalyst stability have become their major drawbacks, so considering the influence of the optimal support and additives is a widely discussed topic nowadays. Unlike traditional oxide supports, the greatest advantage of carbon materials is the weak metal–support interaction. In the above studies, we can draw some general conclusions, as follows: (1) the porous nature and large specific surface area of carbon materials are more conducive to the dispersion of metals and improve the performance and stability of catalysts; (2) the surface of carbon supports presents an inert state, so the dispersion of metal particles can be improved by the introduction of some functional groups (e.g., HNO_3_ treatment, N doping, etc.), which improves the stability of the catalysts; and (3) different preparation methods have a strong influence on the characteristic properties of carbon materials, such as CNT, MC, etc., and exhibit different structures that further affect the reduction of the catalyst, as well as the formation of the active phase.

Research on CO_x_ hydrogenation still focuses on the preparation of carriers and metal loading methods, which are usually inseparable from common methods such as the template method, microwave irradiation method, CVD, etc., while most metal loading still relies on the one-pot method or direct impregnation pyrolysis. In recent years, loading by discharge plasma sintering (SPS) has shown great advantages, especially on CNT carriers, which are sintered at 1200 °C and 30 MPa, where the Fe particles are wrapped by graphite shells and completely reduced, showing high catalytic activity without pre-reduction operation. CNTs have a unique inner and outer surface structure, and their high thermal conductivity facilitates the system’s heat dissipation. Fe particles were embedded in dense CNTs tubes by the SPS technique, and the carbon shells and the skeleton around the CNTs strengthened the reduction of Fe and improved the catalytic activity. In addition, MOFs materials have been gradually applied to CO_x_ hydrogenation reactions in recent years, and their initial structure will gradually disintegrate under high-temperature conditions, which is also favourable for metal particles to be embedded in the carrier and maintain a high specific surface area, which can prolong the catalyst life while obtaining a high activity. While considering multi-metal loading, it seems that we can discuss the case of dual carriers, and it might be good to explore combining the advantages of different materials, for example, through framework support to improve the disadvantage of the low mechanical strength of carbon carriers, so as to optimise the performances of these catalysts and prolong their lifetimes.

Although there have been many studies focusing on the application of carbon-loaded Fe catalysts in CO_x_ hydrogenation reactions, there are still some future challenges, as follows: (1) the low mechanical strength of carbon materials, whether they can continue to maintain the proper size and shape in fixed-bed reactors and under HT-FTS conditions, and how to reduce catalyst wear and product–catalyst separation are also urgent issues to be solved. (2) Weak metal–support interactions, which may affect the reduction and active phase formation. Weak metal–support interactions are more prone to metal sintering during the reaction process, which reduces catalytic activity, so how to use support surface modification to improve the dispersion of metals or additive-induced electronic effects to improve the product distribution should be explored. (3) After the evaluation of the catalytic system is completed or the catalyst is deactivated, is it possible to recycle the metal phases to achieve a net increase in CO_2_ emissions and can the spent catalyst be used as a feedstock for the reaction to produce a product that can be used as a feedstock for the catalyst? Catalysts can be used as a feedstock for the production of syngas.

## Figures and Tables

**Table 1:** Structural parameters of CO hydrogenation catalysts.

Catalyst	Specific Surface Area (m^2^/g)	Pore Volume (cm^3^/g)	Average Pore Size (nm)	Ref.
20Fe/CNTs-Syn	267	1.29	17.4	[48]
Fe/NCNTs-10	110	0.29	10.3	[50]
N-CNT-800	59	0.22	/	[53]
CMK-3-N	1530	1.62	3.5	[61]
Fe/CMK-3S	1326	1.32	3.8	[21]
Fe–C-8	545	0.33	4.3	[64]
Fe/HCS(150)-500	388	0.334	4.4	[94]
Fe/LC-0.2 (800 °C)	404.2	0.09	6.4	[102]
Fe_4_/C_bio_	34.3	0.11	4.3	[95]
Fe@C-F-700	212.9	0.24	4.51	[115]

**Table 2:** Summary of CO hydrogenation catalyst performance.

Catalyst	Pressure (bar)	Temperature (°C)	H_2_/CO	CO Conversion (%)	Selectivity (%)			FTY *	Ref.
					CH_4_	C_2_-C_4_	C_5+_		
Fe_15_Mn_5_-G	15	325	2	92	13.5	/	/	147	[39]
FeK-OX	20	270	1	28.8	19.5	53.6	26.9	/	[46]
20Fe/CNTs-Syn	20	280	1	90.4	54.8	23.6	21.6	313	[48]
Fe/NCNTs-10	20	270	1	45	13.7	9.8	76.5	/	[50]
Fe/N-CNT-h	8	275	2	70.1	/	25.6	60.9	55	[53]
Fe/NCS_ver_	8	275	/	50	22.3	26.1	51.6	/	[24]
Fe/CMK-3S	20	300	2.1	49.7	12.7	39	48.3	340.3	[21]
Fe–C-8	20	270	2	90.1	13.4	/	60.1	/	[64]
15.7 Fe/2 K/AC (2 K)	20.7	270	0.9	41.1	5.7	33.2	61.1	/	[84]
Fe-2MnK-AC	20	320	1	96.8	14.3	32.36	37.4	/	[83]
Fe/HCS(150)-500	20	340	1	80.3	16	31.5	49	/	[94]
Fe_4_/C_bio_	20	300	1	80.9	11.6	22.3	17.5	1198.9	[95]
Fe/NDPC_bio-3_	20	300	1	92	14.6	41.5	43.9	/	[96]
Fe@C-500-Carb	20	230	1	11.4	8.5	22	60.3	40.2	[31]
Fe@C-700	20	300	1	23.15	3.39	11.73	84.88	65	[115]
KFe@C-F300	20	340	1	91.7	7.8	/	/	459	[118]

* FTY, iron time yield, μmolCO·g_Fe_^−1^·s^−1.^

**Table 3:** Structural parameters of CO_2_ hydrogenation catalysts.

Catalyst	Specific Surface Area (m^2^/g)	Pore Volume (cm^3^/g)	Average Pore Size (nm)	Ref.
FeK/MPC	91.22	0.2046	8.1	[66]
FeK/AC	632.55	0.3274	7.7	
20Fe-Co-K/80MMC	496	0.5	4.2	[134]
Fe/LC-0.2 (700 °C)	339.1	0.08	7.18	[102]
Fe@NC-400	595	0.38	/	[152]
Fe/C-K@NC-350-600	42.1	0.06	9.6	[153]

**Table 4:** Summary of CO_2_ hydrogenation catalyst performance.

Catalyst	Pressure (bar)	Temperature (°C)	H_2_/CO_2_	CO_2_ Conversion (%)	Selectivity (%)			FTY *	Ref.
					CH_4_	C_2_-C_4_	C_5+_		
Fe1200SPS	85	350	2	21	34	34	12	113	[131]
FeK/MPC	25	300	3	50.6	/	31.9	44.5	/	[66]
FeK1.5/HSG	20	340	3	/	31	65.9	3.7	73	[98]
Na-Fe@NCNT	15	370	3	48	27.5	52.5	20	/	[132]
GO/K-Fe-Co	30	320	2.5	55.4	13	63.7	23.3	/	[136]
Fe/LC-0.2 (700 °C)	15	320	3	30	/	47	/	/	[102]
FeZnK-NC	30	320	3	34.6	19.1	37.6	22.1	/	[151]
Fe@NC-400	30	320	3	28	26.8	33.8	21.6	4.84	[152]
Fe/C-K@NC-350/600	30	340	3	25.5	16.2	28.4	55.4	/	[153]

* FTY, iron time yield, μmolCO_2_·g_Fe_^−1^·s^−1^.

## References

[1] Ipadeola A.K., Chitt M., Abdelgawad A., Eid K., Abdullah A.M. (2023). Graphene-based catalysts for carbon monoxide oxidation: Experimental and theoretical insights. Int. J. Hydrog. Energy.

[2] Ipadeola A.K., Gamal A., Abdullah A.M., Haruna A.B., Ozoemena K.I., Eid K. (2023). Pd nanocrystals encapsulated in MOF-derived Ni/N-doped hollow carbon nanosheets for efficient thermal CO oxidation: Unveiling the effect of porosity. Catal. Sci. Technol..

[3] Rommens K.T., Saeys M. (2023). Molecular Views on Fischer-Tropsch Synthesis. Chem. Rev..

[4] Yang H.Y., Zhang C., Gao P., Wang H., Li X.P., Zhong L.S., Wei W., Sun Y.H. (2017). A review of the catalytic hydrogenation of carbon dioxide into value-added hydrocarbons. Catal. Sci. Technol..

[5] Mihet M., Dan M., Lazar M.D. (2022). CO_2_ Hydrogenation Catalyzed by Graphene-Based Materials. Molecules.

[6] Puga A.V. (2018). On the nature of active phases and sites in CO and CO_2_ hydrogenation catalysts. Catal. Sci. Technol..

[7] Visconti C.G., Martinelli M., Falbo L., Fratalocchi L., Lietti L. (2016). CO_2_ hydrogenation to hydrocarbons over Co and Fe-based Fischer-Tropsch catalysts. Catal. Today.

[8] Arizapana K., Schossig J., Wildy M., Weber D., Gandotra A., Jayaraman S., Wei W.Y., Xu K., Yu L., Mugweru A.M. (2024). Harnessing the Synergy of Fe and Co with Carbon Nanofibers for Enhanced CO_2_ Hydrogenation Performance. ACS Sustain. Chem. Eng..

[9] Oschatz M., Lamme W.S., Xie J.X., Dugulan A.I., de Jong K.P. (2016). Ordered Mesoporous Materials as Supports for Stable Iron Catalysts in the Fischer-Tropsch Synthesis of Lower Olefins. ChemCatChem.

[10] Liu J.X., Wang P., Xu W., Hensen E.J.M. (2017). Particle Size and Crystal Phase Effects in Fischer-Tropsch Catalysts. Engineering.

[11] Zhu J., Zhang G.H., Li W.H., Zhang X.B., Ding F.S., Song C.S., Guo X.W. (2020). Deconvolution of the Particle Size Effect on CO_2_ Hydrogenation over Iron-Based Catalysts. ACS Catal..

[12] Yahyazadeh A., Borugadda V.B., Dalai A.K., Zhang L.F. (2022). Optimization of olefins’ yield in Fischer-Tropsch synthesis using carbon nanotubes supported iron catalyst with potassium and molybdenum promoters. Appl. Catal. A-Gen..

[13] Dad M., Lancee R.J., van Vuuren M.J., van de Loosdrecht J., Niemantsverdriet J.W.H., Fredriksson H.O.A. (2017). SiO_2_-supported Fe & FeMn colloids-Fischer-Tropsch synthesis on 3D model catalysts. Appl. Catal. A-Gen..

[14] Abrokwah R.Y., Rahman M.M., Deshmane V.G., Kuila D. (2019). Effect of titania support on Fischer-Tropsch synthesis using cobalt, iron, and ruthenium catalysts in silicon-microchannel microreactor. Mol. Catal..

[15] Ali S., Zabidi N.A.M., Al-Marri M.J., Khader M.M. (2017). Effect of the support on physicochemical properties and catalytic performance of cobalt based nano-catalysts in Fischer-Tropsch reaction. Mater. Today Commun..

[16] Munirathinam R., Minh D.P., Nzihou A. (2018). Effect of the Support and Its Surface Modifications in Cobalt-Based Fischer-Tropsch Synthesis. Ind. Eng. Chem. Res..

[17] Dlamini M.W., Phaahlamohlaka T.N., Kumi D.O., Forbes R., Jewell L.L., Coville N.J. (2020). Post doped nitrogen-decorated hollow carbon spheres as a support for Co Fischer-Tropsch catalysts. Catal. Today.

[18] Asalieva E., Sineva L., Sinichkina S., Solomonik I., Gryaznov K., Pushina E., Kulchakovskaya E., Gorshkov A., Kulnitskiy B., Ovsyannikov D. (2020). Exfoliated graphite as a heat-conductive frame for a new pelletized Fischer-Tropsch synthesis catalyst. Appl. Catal. A-Gen..

[19] Galvis H.M.T., Bitter J.H., Khare C.B., Ruitenbeek M., Dugulan A.I., de Jong K.P. (2012). Supported Iron Nanoparticles as Catalysts for Sustainable Production of Lower Olefins. Science.

[20] Guo L.S., Zhang P.P., Cui Y., Liu G.B., Wu J.H., Yang G.H., Yoneyama Y., Tsubaki N. (2019). One-Pot Hydrothermal Synthesis of Nitrogen Functionalized Carbonaceous Material Catalysts with Embedded Iron Nanoparticles for CO_2_ Hydrogenation. ACS Sustain. Chem. Eng..

[21] Cheng K., Ordomsky V.V., Virginie M., Legras B., Chernavskii P.A., Kazak V.O., Cordier C., Paul S., Wang Y., Khodakov A.Y. (2014). Support effects in high temperature Fischer-Tropsch synthesis on iron catalysts. Appl. Catal. A-Gen..

[22] Sun B., Xu K., Nguyen L., Qiao M.H., Tao F. (2012). Preparation and Catalysis of Carbon-Supported Iron Catalysts for Fischer-Tropsch Synthesis. ChemCatChem.

[23] Xiong H.F., Jewell L.L., Coville N.J. (2015). Shaped Carbons As Supports for the Catalytic Conversion of Syngas to Clean Fuels. ACS Catal..

[24] Xiong H., Moyo M., Motchelaho M.A., Tetana Z.N., Dube S.M.A., Jewell L.L., Coville N.J. (2014). Fischer–Tropsch synthesis: Iron catalysts supported on N-doped carbon spheres prepared by chemical vapor deposition and hydrothermal approaches. J. Catal..

[25] Chen H.C., Sun F.G., Wang J.T., Li W.C., Qiao W.M., Ling L.C., Long D.H. (2013). Nitrogen Doping Effects on the Physical and Chemical Properties of Mesoporous Carbons. J. Phys. Chem. C.

[26] Liu G., Chen Q., Oyunkhand E., Ding S., Yamane N., Yang G., Yoneyama Y., Tsubaki N. (2018). Nitrogen-rich mesoporous carbon supported iron catalyst with superior activity for Fischer-Tropsch synthesis. Carbon.

[27] Deng C., Xu L.J., Hu K.H., Chen X.X., Gao R.X., Zhang L.Y., Wang L., Zhang C.D. (2023). Research Advances on Nitrogen-Doped Carbon Materials in CO_x_ Hydrogenation. Atmosphere.

[28] Evdokimenko N.D., Kapustin G.I., Tkachenko O.P., Kalmykov K.B., Kustov A.L. (2022). Zn Doping Effect on the Performance of Fe-Based Catalysts for the Hydrogenation of CO_2_ to Light Hydrocarbons. Molecules.

[29] Amin M., Usman M., Kella T., Khan W.U., Khan I.A., Lee K.H. (2024). Issues and challenges of Fischer-Tropsch synthesis catalysts. Front. Chem..

[30] van Deelen T.W., Mejía C.H., de Jong K.P. (2019). Control of metal-support interactions in heterogeneous catalysts to enhance activity and selectivity. Nat. Catal..

[31] Wezendonk T.A., Sun X., Dugulan A.I., van Hoof A.J.F., Hensen E.J.M., Kapteijn F., Gascon J. (2018). Controlled formation of iron carbides and their performance in Fischer-Tropsch synthesis. J. Catal..

[32] Dry M.E. (2002). The Fischer-Tropsch process: 1950–2000. Catal. Today.

[33] Huber G.W., Iborra S., Corma A. (2006). Synthesis of transportation fuels from biomass: Chemistry, catalysts, and engineering. Chem. Rev..

[34] Liu R.J., Xu Y., Qiao Y., Li Z.H., Ma X.B. (2015). Factors influencing the Fischer-Tropsch synthesis performance of iron-based catalyst: Iron oxide dispersion, distribution and reducibility. Fuel Process. Technol..

[35] Valero-Romero M.J., Rodríguez-Cano M.A., Palomo J., Rodríguez-Mirasol J., Cordero T. (2021). Carbon-Based Materials as Catalyst Supports for Fischer-Tropsch Synthesis: A Review. Front. Mater..

[36] Wan H.J., Wu B.S., Xiang H.W., Li Y.W. (2012). Fischer-Tropsch Synthesis: Influence of Support Incorporation Manner on Metal Dispersion, Metal-Support Interaction, and Activities of Iron Catalysts. ACS Catal..

[37] Zhang G.J., Qu J.W., Du Y.N., Guo F.B., Zhao H.X., Zhang Y.F., Xu Y. (2014). Hydrogen production from CO_2_ reforming of methane over high pressure H_2_O_2_ modified different semi-cokes. J. Ind. Eng. Chem..

[38] De Jong K.P., Geus J.W. (2000). Carbon nanofibers: Catalytic synthesis and applications. Catal. Rev.-Sci. Eng..

[39] Moussa S.O., Panchakarla L.S., Ho M.Q., El-Shall M.S. (2014). Graphene-Supported, Iron-Based Nanoparticles for Catalytic Production of Liquid Hydrocarbons from Synthesis Gas: The Role of the Graphene Support in Comparison with Carbon Nanotubes. ACS Catal..

[40] Serp P., Corrias M., Kalck P. (2003). Carbon nanotubes and nanofibers in catalysis. Appl. Catal. A-Gen..

[41] Ajayan P.M. (1999). Nanotubes from carbon. Chem. Rev..

[42] Chen W., Fan Z.L., Pan X.L., Bao X.H. (2008). Effect of confinement in carbon nanotubes on the activity of Fischer-Tropsch iron catalyst. J. Am. Chem. Soc..

[43] Guczi L., Stefler G., Geszti O., Koppány Z., Kónya Z., Molnár É., Urbán M., Kiricsi I. (2006). CO hydrogenation over cobalt and iron catalysts supported over multiwall carbon nanotubes: Effect of preparation. J. Catal..

[44] Xiong H.F., Motchelaho M.A.M., Moyo M., Jewell L.L., Coville N.J. (2013). Cobalt catalysts supported on a micro-coil carbon in Fischer-Tropsch synthesis: A comparison with CNTs and CNFs. Catal. Today.

[45] Xiong H.F., Motchelaho M.A.M., Moyo M., Jewell L.L., Coville N.J. (2011). Correlating the preparation and performance of cobalt catalysts supported on carbon nanotubes and carbon spheres in the Fischer-Tropsch synthesis. J. Catal..

[46] Duan X., Wang D., Qian G., Walmsley J.C., Holmen A., Chen D., Zhou X. (2016). Fabrication of K-promoted iron/carbon nanotubes composite catalysts for the Fischer–Tropsch synthesis of lower olefins. J. Energy Chem..

[47] Kundu S., Wang Y.M., Xia W., Muhler M. (2008). Thermal Stability and Reducibility of Oxygen-Containing Functional Groups on Multiwalled Carbon Nanotube Surfaces: A Quantitative High-Resolution XPS and TPD/TPR Study. J. Phys. Chem. C.

[48] Yahyazadeh A., Borugadda V.B., Dalai A.K., Zhang L.F. (2022). Optimization of carbon nanotube growth via response surface methodology for Fischer-Tropsch synthesis over Fe/CNT catalyst. Catal. Today.

[49] Fang Y., Cao J., Zhang X., Cao Y., Song N., Qian G., Zhou X., Duan X. (2021). Crucial roles of support modification and promoter introduction in Fe/CNT catalyzed syngas conversion to lower olefins. Catal. Today.

[50] Li Z., Liu R., Xu Y., Ma X. (2015). Enhanced Fischer–Tropsch synthesis performance of iron-based catalysts supported on nitric acid treated N-doped CNTs. Appl. Surf. Sci..

[51] Chew L.M., Kangvansura P., Ruland H., Schulte H.J., Somsen C., Xia W., Eggeler G., Worayingyong A., Muhler M. (2014). Effect of nitrogen doping on the reducibility, activity and selectivity of carbon nanotube-supported iron catalysts applied in CO_2_ hydrogenation. Appl. Catal. A Gen..

[52] Fu T.J., Lv J., Li Z.H. (2014). Effect of Carbon Porosity and Cobalt Particle Size on the Catalytic Performance of Carbon Supported Cobalt Fischer-Tropsch Catalysts. Ind. Eng. Chem. Res..

[53] Xiong H., Motchelaho M.A., Moyo M., Jewell L.L., Coville N.J. (2014). Fischer–Tropsch synthesis: Iron-based catalysts supported on nitrogen-doped carbon nanotubes synthesized by post-doping. Appl. Catal. A Gen..

[54] Chew L.M., Xia W., Düdder H., Weide P., Ruland H., Muhler M. (2016). On the role of the stability of functional groups in multi-walled carbon nanotubes applied as support in iron-based high-temperature Fischer-Tropsch synthesis. Catal. Today.

[55] Wu D.C., Fu R.W., Dresselhaus M.S., Dresselhaus G. (2006). Fabrication and nano-structure control of carbon aerogels via a microemulsion-templated sol-gel polymerization method. Carbon.

[56] Enterría M., Figueiredo J.L. (2016). Nanostructured mesoporous carbons: Tuning texture and surface chemistry. Carbon.

[57] Li W., Yue Q., Deng Y.H., Zhao D.Y. (2013). Ordered Mesoporous Materials Based on Interfacial Assembly and Engineering. Adv. Mater..

[58] Liang C.D., Li Z.J., Dai S. (2008). Mesoporous carbon materials: Synthesis and modification. Angew. Chem.-Int. Ed..

[59] Long D.H., Zhang J., Yang J.H., Hu Z.J., Cheng G., Liu X.M., Zhang R., Zhan L., Qiao W.M., Ling L.C. (2008). Chemical state of nitrogen in carbon aerogels issued from phenol-melamine-formaldehyde gels. Carbon.

[60] Lee J.S., Joo S.H., Ryoo R. (2002). Synthesis of mesoporous silicas of controlled pore wall thickness and their replication to ordered nanoporous carbons with various pore diameters. J. Am. Chem. Soc..

[61] Oschatz M., Hofmann J.P., van Deelen T.W., Lamme W.S., Krans N.A., Hensen E.J.M., de Jong K.P. (2017). Effects of the Functionalization of the Ordered Mesoporous Carbon Support Surface on Iron Catalysts for the Fischer–Tropsch Synthesis of Lower Olefins. ChemCatChem.

[62] Wu Z.X., Webley P.A., Zhao D.Y. (2010). Comprehensive Study of Pore Evolution, Mesostructural Stability, and Simultaneous Surface Functionalization of Ordered Mesoporous Carbon (FDU-15) by Wet Oxidation as a Promising Adsorbent. Langmuir.

[63] Zhai Y.P., Dou Y.Q., Zhao D.Y., Fulvio P.F., Mayes R.T., Dai S. (2011). Carbon Materials for Chemical Capacitive Energy Storage. Adv. Mater..

[64] Sun Z., Sun B., Qiao M., Wei J., Yue Q., Wang C., Deng Y., Kaliaguine S., Zhao D. (2012). A General Chelate-Assisted Co-Assembly to Metallic Nanoparticles-Incorporated Ordered Mesoporous Carbon Catalysts for Fischer–Tropsch Synthesis. J. Am. Chem. Soc..

[65] Chen X.Q., Deng D.H., Pan X.L., Hu Y.F., Bao X.H. (2015). N-doped graphene as an electron donor of iron catalysts for CO hydrogenation to light olefins. Chem. Commun..

[66] Hwang S.-M., Zhang C., Han S.J., Park H.-G., Kim Y.T., Yang S., Jun K.-W., Kim S.K. (2020). Mesoporous carbon as an effective support for Fe catalyst for CO_2_ hydrogenation to liquid hydrocarbons. J. CO2 Util..

[67] Cheng Y., Fan Y.Q., Pei Y., Qiao M.H. (2015). Graphene-supported metal/metal oxide nanohybrids: Synthesis and applications in heterogeneous catalysis. Catal. Sci. Technol..

[68] Rao C.N.R., Sood A.K., Subrahmanyam K.S., Govindaraj A. (2009). Graphene: The New Two-Dimensional Nanomaterial. Angew. Chem.-Int. Ed..

[69] Allen M.J., Tung V.C., Kaner R.B. (2010). Honeycomb Carbon: A Review of Graphene. Chem. Rev..

[70] Su C.Y., Lu A.Y., Xu Y.P., Chen F.R., Khlobystov A.N., Li L.J. (2011). High-Quality Thin Graphene Films from Fast Electrochemical Exfoliation. ACS Nano.

[71] Shang N.G., Papakonstantinou P., McMullan M., Chu M., Stamboulis A., Potenza A., Dhesi S.S., Marchetto H. (2008). Catalyst-Free Efficient Growth, Orientation and Biosensing Properties of Multilayer Graphene Nanoflake Films with Sharp Edge Planes. Adv. Funct. Mater..

[72] Dreyer D.R., Park S., Bielawski C.W., Ruoff R.S. (2010). The chemistry of graphene oxide. Chem. Soc. Rev..

[73] Dreyer D.R., Todd A.D., Bielawski C.W. (2014). Harnessing the chemistry of graphene oxide. Chem. Soc. Rev..

[74] Loh K.P., Bao Q.L., Ang P.K., Yang J.X. (2010). The chemistry of graphene. J. Mater. Chem..

[75] Zhang X.L., Lu Z.S., Xu G.L., Wang T.X., Ma D.W., Yang Z.X., Yang L. (2015). Single Pt atom stabilized on nitrogen doped graphene: CO oxidation readily occurs via the tri-molecular Eley-Rideal mechanism. Phys. Chem. Chem. Phys..

[76] Fampiou I., Ramasubramaniam A. (2012). Binding of Pt Nanoclusters to Point Defects in Graphene: Adsorption, Morphology, and Electronic Structure. J. Phys. Chem. C.

[77] Okamoto Y. (2006). Density-functional calculations of icosahedral M_13_ (M = Pt and Au) clusters on graphene sheets and flakes. Chem. Phys. Lett..

[78] Wang X.R., Li X.L., Zhang L., Yoon Y., Weber P.K., Wang H.L., Guo J., Dai H.J. (2009). N-Doping of Graphene Through Electrothermal Reactions with Ammonia. Science.

[79] Huang S.F., Terakura K., Ozaki T., Ikeda T., Boero M., Oshima M., Ozaki J., Miyata S. (2009). First-principles calculation of the electronic properties of graphene clusters doped with nitrogen and boron: Analysis of catalytic activity for the oxygen reduction reaction. Phys. Rev. B.

[80] Xu L.Y., Wang Q.X., Xu Y.D., Huang J.S. (1995). Promotion effect of K_2_O and MnO additives on the selective production of light alkenes via syngas over Fe/silicalite-2 catalysts. Catal. Lett..

[81] Zhang J.L., Fan S.B., Zhao T.S., Li W.H., Sun Y.H. (2011). Carbon modified Fe-Mn-K catalyst for the synthesis of light olefins from CO hydrogenation. React. Kinet. Mech. Catal..

[82] Hassan H.M.A., Abdelsayed V., Khder A., AbouZeid K.M., Terner J., El-Shall M.S., Al-Resayes S.I., El-Azhary A.A. (2009). Microwave synthesis of graphene sheets supporting metal nanocrystals in aqueous and organic media. J. Mater. Chem..

[83] Tian Z., Wang C., Si Z., Ma L., Chen L., Liu Q., Zhang Q., Huang H. (2017). Fischer-Tropsch synthesis to light olefins over iron-based catalysts supported on KMnO_4_ modified activated carbon by a facile method. Appl. Catal. A Gen..

[84] Ma W., Kugler E.L., Dadyburjor D.B. (2007). Potassium Effects on Activated-Carbon-Supported Iron Catalysts for Fischer−Tropsch Synthesis. Energy Fuels.

[85] Arakawa H., Bell A.T. (2002). Effects of potassium promotion on the activity and selectivity of iron Fischer-Tropsch catalysts. Ind. Eng. Chem. Process Des. Dev..

[86] Campos A., Lohitharn N., Roy A., Lotero E., Goodwin J.G., Spivey J.J. (2010). An activity and XANES study of Mn-promoted, Fe-based Fischer-Tropsch catalysts. Appl. Catal. A-Gen..

[87] Zhang Q.H., Kang J.C., Wang Y. (2010). Development of Novel Catalysts for Fischer-Tropsch Synthesis: Tuning the Product Selectivity. ChemCatChem.

[88] Tian Z.P., Wang C.G., Si Z., Wen C.Y., Xu Y., Lv W., Chen L.G., Zhang X.H., Ma L.L. (2019). Enhancement of Light Olefins Selectivity Over N-Doped Fischer-Tropsch Synthesis Catalyst Supported on Activated Carbon Pretreated with KMnO_4_. Catalysts.

[89] Asami K., Komiyama K., Yoshida K., Miyahara H. (2018). Synthesis of Lower Olefins from Synthesis Gas over Active Carbon-Supported Iron Catalyst. Catal. Today.

[90] Sevilla M., Fuertes A.B. (2009). Chemical and Structural Properties of Carbonaceous Products Obtained by Hydrothermal Carbonization of Saccharides. Chem.-A Eur. J..

[91] Sun X.M., Li Y.D. (2004). Ga_2_O_3_ and GaN semiconductor hollow spheres. Angew. Chem.-Int. Ed..

[92] Yu G., Sun B., Pei Y., Xie S., Yan S., Qiao M., Fan K., Zhang X., Zong B. (2009). FexOy@C Spheres as an Excellent Catalyst for Fischer−Tropsch Synthesis. J. Am. Chem. Soc..

[93] Xiong H., Moyo M., Motchelaho M.A.M., Jewell L.L., Coville N.J. (2010). Fischer–Tropsch synthesis over model iron catalysts supported on carbon spheres: The effect of iron precursor, support pretreatment, catalyst preparation method and promoters. Appl. Catal. A Gen..

[94] Teng X.S., Huang S.Y., Wang J., Wang H.Y., Zhao Q., Yuan Y., Ma X.B. (2018). Fabrication of Fe_2_C Embedded in Hollow Carbon Spheres: A High-Performance and Stable Catalyst for Fischer-Tropsch Synthesis. ChemCatChem.

[95] Bai J., Qin C., Xu Y., Du Y., Ma G., Ding M. (2021). Biosugarcane-based carbon support for high-performance iron-based Fischer-Tropsch synthesis. iScience.

[96] Bai J., Qin C., Xu Y., Xu D., Ding M. (2022). Preparation of Nitrogen Doped Biochar-Based Iron Catalyst for Enhancing Gasoline-Range Hydrocarbons Production. ACS Appl. Mater. Interfaces.

[97] Amin M., Munir S., Iqbal N., Wabaidur S., Iqbal A. (2022). The Conversion of Waste Biomass into Carbon-Supported Iron Catalyst for Syngas to Clean Liquid Fuel Production. Catalysts.

[98] Wu T., Lin J., Cheng Y., Tian J., Wang S., Xie S., Pei Y., Yan S., Qiao M., Xu H. (2018). Porous Graphene-Confined Fe–K as Highly Efficient Catalyst for CO_2_ Direct Hydrogenation to Light Olefins. ACS Appl. Mater. Interfaces.

[99] Chernyak S., Burtsev A., Maksimov S., Kupreenko S., Maslakov K., Savilov S. (2020). Structural evolution, stability, deactivation and regeneration of Fischer-Tropsch cobalt-based catalysts supported on carbon nanotubes. Appl. Catal. A-Gen..

[100] Lin B.Y., Guo Y.J., Lin J.D., Ni J., Lin J.X., Jiang L.L., Wang Y. (2017). Deactivation study of carbon-supported ruthenium catalyst with potassium promoter. Appl. Catal. A-Gen..

[101] Chakraborty R., Vilya K., Pradhan M., Nayak A.K. (2022). Recent advancement of biomass-derived porous carbon based materials for energy and environmental remediation applications. J. Mater. Chem. A.

[102] Zhu R., Wang K., Xing Y., Li C., Gao X., Ma Q., Zhao T.-s., Zhang J. (2024). Preparation of Fe-based catalysts from waste biomass as a carbon carrier and its catalytic performance in CO_2_ hydrogenation. New J. Chem..

[103] Li S.Z., Meitzner G.D., Iglesia E. (2001). Structure and site evolution of iron oxide catalyst precursors during the Fischer-Tropsch synthesis. J. Phys. Chem. B.

[104] de Smit E., Beale A.M., Nikitenko S., Weckhuysen B.M. (2009). Local and long range order in promoted iron-based Fischer-Tropsch catalysts: A combined in situ X-ray absorption spectroscopy/wide angle X-ray. J. Catal..

[105] Herranz T., Rojas S., Pérez-Alonso F.J., Ojeda M., Terreros P., Fierro J.L.G. (2006). Genesis of iron carbides and their role in the synthesis of hydrocarbons from synthesis gas. J. Catal..

[106] Lyu S., Wang L., Li Z., Yin S.K., Chen J., Zhang Y.H., Li J.L., Wang Y. (2020). Stabilization of ε-iron carbide as high-temperature catalyst under realistic Fischer-Tropsch synthesis conditions. Nat. Commun..

[107] Xu K., Sun B., Lin J., Wen W., Pei Y., Yan S.R., Qiao M.H., Zhang X.X., Zong B.N. (2014). ε-Iron carbide as a low-temperature Fischer-Tropsch synthesis catalyst. Nat. Commun..

[108] Zhao Q., Liang H.T., Huang S.Y., Han X.X., Wang H.Y., Wang J., Wang Y., Ma X.B. (2021). Tunable Fe_3_O_4_ nanoparticles assembled porous microspheres as catalysts for Fischer-Tropsch synthesis to lower olefins. Catal. Today.

[109] Shetty A., Molahalli V., Sharma A., Hegde G. (2023). Biomass-Derived Carbon Materials in Heterogeneous Catalysis: A Step towards Sustainable Future. Catalysts.

[110] Stepacheva A.A., Markova M.E., Lugovoy Y.V., Kosivtsov Y.Y., Matveeva V.G., Sulman M.G. (2023). Plant-Biomass-Derived Carbon Materials as Catalyst Support, A Brief Review. Catalysts.

[111] Otun K.O., Liu X., Hildebrandt D. (2020). Metal-organic framework (MOF)-derived catalysts for Fischer-Tropsch synthesis: Recent progress and future perspectives. J. Energy Chem..

[112] Razavi S.A.A., Morsali A. (2019). Linker functionalized metal-organic frameworks. Coord. Chem. Rev..

[113] Chen L.Y., Xu Q. (2019). Metal-Organic Framework Composites for Catalysis. Matter.

[114] Wezendonk T.A., Santos V.P., Nasalevich M.A., Warringa Q.S.E., Dugulan A.I., Chojecki A., Koeken A.C.J., Ruitenbeek M., Meima G., Islam H.U. (2016). Elucidating the Nature of Fe Species during Pyrolysis of the Fe-BTC MOF into Highly Active and Stable Fischer-Tropsch Catalysts. ACS Catal..

[115] Nisa M.U., Chen Y., Li X., Jiang X., Li Z. (2022). Modulating C_5+_selectivity for Fischer-Tropsch synthesis by tuning pyrolysis temperature of MOFs derived Fe-based catalyst. J. Taiwan Inst. Chem. Eng..

[116] Abbaslou R.M.M., Soltan J., Dalai A.K. (2010). Effects of nanotubes pore size on the catalytic performances of iron catalysts supported on carbon nanotubes for Fischer-Tropsch synthesis. Appl. Catal. A-Gen..

[117] An B., Cheng K., Wang C., Wang Y., Lin W.B. (2016). Pyrolysis of Metal-Organic Frameworks to Fe_3_O_4_@Fe_5_C_2_ Core-Shell Nanoparticles for Fischer-Tropsch Synthesis. ACS Catal..

[118] Wezendonk T.A., Warringa Q.S.E., Santos V.P., Chojecki A., Ruitenbeek M., Meima G., Makkee M., Kapteijn F., Gascon J. (2017). Structural and elemental influence from various MOFs on the performance of Fe@C catalysts for Fischer–Tropsch synthesis. Faraday Discuss..

[119] Cho J.M., Kim B.G., Han G.Y., Sun J., Jeong H.K., Bae J.W. (2020). Effects of metal-organic framework-derived iron carbide phases for CO hydrogenation activity to hydrocarbons. Fuel.

[120] Santos V.P., Wezendonk T.A., Jaén J.J.D., Dugulan A.I., Nasalevich M.A., Islam H.U., Chojecki A., Sartipi S., Sun X., Hakeem A.A. (2015). Metal organic framework-mediated synthesis of highly active and stable Fischer-Tropsch catalysts. Nat. Commun..

[121] Lippi R., Howard S.C., Barron H., Easton C.D., Madsen I.C., Waddington L.J., Vogt C., Hill M.R., Sumby C.J., Doonan C.J. (2017). Highly active catalyst for CO_2_ methanation derived from a metal organic framework template. J. Mater. Chem. A.

[122] Ye R.P., Ding J., Gong W.B., Argyle M.D., Zhong Q., Wang Y.J., Russell C.K., Xu Z.H., Russell A.G., Li Q.H. (2019). CO_2_ hydrogenation to high-value products via heterogeneous catalysis. Nat. Commun..

[123] Yao B.Z., Xiao T.C., Makgae O.A., Jie X.Y., Gonzalez-Cortes S., Guan S.L., Kirkland A.I., Dilworth J.R., Al-Megren H.A., Alshihri S.M. (2020). Transforming carbon dioxide into jet fuel using an organic combustion-synthesized Fe-Mn-K catalyst. Nat. Commun..

[124] Liu J.T., Zhang Y.C., Peng C. (2024). Recent Advances Hydrogenation of Carbon Dioxide to Light Olefins over Iron-Based Catalysts via the Fischer-Tropsch Synthesis. ACS Omega.

[125] Wang D., Xie Z.H., Porosoff M.D., Chen J.G.G. (2021). Recent advances in carbon dioxide hydrogenation to produce olefins and aromatics. Chem.

[126] Liu Y.H., Cheng Q.P., Xiong S.H., Zhang Y.T., Tan L., Song S., Ding T., Tian Y., Li X.G. (2025). Enhancing CO_2_ hydrogenation performance via the synergistic effects of iron carbides and iron oxides. Int. J. Hydrogen Energy.

[127] Meng W.X., de Jong B.C.A., van de Bovenkamp H., Boer G.J., Bezemer G.L., Dugulan A.I., Xie J.X. (2024). Selectivity control between reverse water-gas shift and fischer-tropsch synthesis in carbon-supported iron-based catalysts for CO_2_ hydrogenation. Chem. Eng. J..

[128] Minett D.R., O’Byrne J.P., Pascu S.I., Plucinski P.K., Owen R.E., Jones M.D., Mattia D. (2014). Fe@CNT-monoliths for the conversion of carbon dioxide to hydrocarbons: Structural characterisation and Fischer-Tropsch reactivity investigations. Catal. Sci. Technol..

[129] Suslova E., Savilov S., Egorov A., Shumyantsev A., Lunin V. (2020). Carbon nanotube frameworks by spark plasma sintering. Microporous Mesoporous Mater..

[130] Chernyak S.A., Ivanov A.S., Maksimov S.V., Maslakov K.I., Isaikina O.Y., Chernavskii P.A., Kazantsev R.V., Eliseev O.L., Savilov S.S. (2020). Fischer-Tropsch synthesis over carbon-encapsulated cobalt and iron nanoparticles embedded in 3D-framework of carbon nanotubes. J. Catal..

[131] Chernyak S.A., Ivanov A.S., Stolbov D.N., Maksimov S.V., Maslakov K.I., Chernavskii P.A., Pokusaeva Y.A., Koklin A.E., Bogdan V.I., Savilov S.V. (2020). Sintered Fe/CNT framework catalysts for CO_2_ hydrogenation into hydrocarbons. Carbon.

[132] Williamson D.L., Herdes C., Torrente-Murciano L., Jones M.D., Mattia D. (2019). N-Doped Fe@CNT for Combined RWGS/FT CO_2_ Hydrogenation. ACS Sustain. Chem. Eng..

[133] Landau M.V., Meiri N., Utsis N., Nehemya R.V., Herskowitz M. (2017). Conversion of CO_2_, CO, and H_2_ in CO_2_ Hydrogenation to Fungible Liquid Fuels on Fe-Based Catalysts. Ind. Eng. Chem. Res..

[134] Witoon T., Numpilai T., Nueangnoraj K., Cheng C.K., Chareonpanich M., Limtrakul J. (2022). Light olefins synthesis from CO_2_ hydrogenation over mixed Fe-Co-K supported on micro-mesoporous carbon catalysts. Int. J. Hydrogen Energy.

[135] Peng L., Jurca B., Primo A., Gordillo A., Parvulescu V.I., García H. (2021). Co–Fe Clusters Supported on N-Doped Graphitic Carbon as Highly Selective Catalysts for Reverse Water Gas Shift Reaction. ACS Sustain. Chem. Eng..

[136] Liang J.M., Liu J.T., Guo L.S., Wang W.H., Wang C.W., Gao W.Z., Guo X.Y., He Y.L., Yang G.H., Yasuda S. (2024). CO_2_ hydrogenation over Fe-Co bimetallic catalysts with tunable selectivity through a graphene fencing approach. Nat. Commun..

[137] Wang H., Sun K., Tao F., Stacchiola D.J., Hu Y.H. (2013). 3D Honeycomb-Like Structured Graphene and Its High Efficiency as a Counter-Electrode Catalyst for Dye-Sensitized Solar Cells. Angew. Chem.-Int. Ed..

[138] Felgueiras M.B.S., Pereira M.F.R., Soares O. (2024). Effect of the support on the CO_2_ hydrogenation to C_2_-C_4_ products. Catal. Today.

[139] Chen J.Y., Han S.J., Park H.G., Nasriddinov K., Zhang C.D., Jun K.W., Kim S.K. (2023). Benchmarking promoters of Fe/activated carbon catalyst for stable hydrogenation of CO_2_ to liquid hydrocarbons. Appl. Catal. B-Environ. Energy.

[140] Svidersky S.A., Dement’eva O.S., Ivantsov M.I., Grabchak A.A., Kulikova M.V., Maximov A.L. (2023). Hydrogenation of CO_2_ over Biochar-Supported Catalysts. Pet. Chem..

[141] Niu L.W., Liu X.W., Wen X.D., Yang Y., Xu J., Li Y.W. (2020). Effect of potassium promoter on phase transformation during H_2_ pretreatment of a Fe_2_O_3_ Fischer Tropsch synthesis catalyst precursor. Catal. Today.

[142] Amoyal M., Vidruk-Nehemya R., Landau M.V., Herskowitz M. (2017). Effect of potassium on the active phases of Fe catalysts for carbon dioxide conversion to liquid fuels through hydrogenation. J. Catal..

[143] Hu S., Liu M., Ding F., Song C., Zhang G., Guo X. (2016). Hydrothermally stable MOFs for CO_2_ hydrogenation over iron-based catalyst to light olefins. J. CO2 Util..

[144] Assfour B., Leoni S., Seifert G. (2010). Hydrogen Adsorption Sites in Zeolite Imidazolate Frameworks ZIF-8 and ZIF-11. J. Phys. Chem. C.

[145] Wu H., Zhou W., Yildirim T. (2007). Hydrogen storage in a prototypical zeolitic imidazolate framework-8. J. Am. Chem. Soc..

[146] Ramirez A., Gevers L., Bavykina A., Ould-Chikh S., Gaston J. (2018). Metal Organic Framework-Derived Iron Catalysts for the Direct Hydrogenation of CO_2_ to Short Chain Olefins. ACS Catal..

[147] Feng S.S., Li W., Shi Q., Li Y.H., Chen J.C., Ling Y., Asiri A.M., Zhao D.Y. (2014). Synthesis of nitrogen-doped hollow carbon nanospheres for CO_2_ capture. Chem. Commun..

[148] Wei J., Zhou D.D., Sun Z.K., Deng Y.H., Xia Y.Y., Zhao D.Y. (2013). A Controllable Synthesis of Rich Nitrogen-Doped Ordered Mesoporous Carbon for CO_2_ Capture and Supercapacitors. Adv. Funct. Mater..

[149] Li Y.H., Hung T.H., Chen C.W. (2009). A first-principles study of nitrogen- and boron-assisted platinum adsorption on carbon nanotubes. Carbon.

[150] He L., Weniger F., Neumann H., Beller M. (2016). Synthesis, Characterization, and Application of Metal Nanoparticles Supported on Nitrogen-Doped Carbon: Catalysis beyond Electrochemistry. Angew. Chem.-Int. Ed..

[151] Liu J., Sun Y., Jiang X., Zhang A., Song C., Guo X. (2018). Pyrolyzing ZIF-8 to N-doped porous carbon facilitated by iron and potassium for CO_2_ hydrogenation to value-added hydrocarbons. J. CO2 Util..

[152] Liu J., Zhang A., Jiang X., Zhang G., Sun Y., Liu M., Ding F., Song C., Guo X. (2019). Overcoating the Surface of Fe-Based Catalyst with ZnO and Nitrogen-Doped Carbon toward High Selectivity of Light Olefins in CO_2_ Hydrogenation. Ind. Eng. Chem. Res..

[153] Xu F., Meng X., Zhao R., Jin D.M., Dai W.H., Xu B.W., Yang D.D., Xin Z. (2024). Metal-organic framework-derived Fe_3_O_4_-FeC_x_ catalyst for direct CO_2_ hydrogenation to light olefins. Appl. Catal. A-Gen..

